# WTAP regulates NCOA4-mediated ferroptosis via a YTHDF2-dependent mechanism in preeclampsia

**DOI:** 10.1186/s13148-025-02004-w

**Published:** 2025-11-19

**Authors:** Can Li, Zhiyuan Li, Chunling Ma, Lin Xu, Ning Zhang, Yan Li, Qingqing Lv, Chao Li, Shuping Zhao

**Affiliations:** 1 Department of Obstetrics and Gynecology, The Affiliated Hospital of Qingdao University, Qingdao, 266003 Shandong Province China; 2 Department of Medical Research Center, The Affiliated Hospital of Qingdao University, Qingdao, 266003 Shandong Province China; 3 Department of Obstetrics and Gynecology, Qingdao Women and Children’s Hospital, Qingdao, 266111 Shandong Province China

**Keywords:** Preeclampsia, WTAP, NCOA4, Ferroptosis, M^6^A methyladenosine

## Abstract

**Background:**

Preeclampsia (PE) is a pregnancy-specific hypertensive disorder associated with placental dysfunction and oxidative stress. This study explored whether WTAP regulates ferroptosis in trophoblasts through m^6^A-dependent control of NCOA4 and YTHDF2.

**Methods:**

WTAP expression and global m^6^A levels in PE placentas were examined by qRT-PCR, western blot, and immunohistochemistry, along with histopathological analysis. WTAP, NCOA4, and YTHDF2 expression were manipulated in HTR-8/SVneo trophoblasts using siRNAs or overexpression plasmids. Cell proliferation, migration, cell-cycle distribution, oxidative stress, and ferroptosis markers were evaluated. MeRIP-qPCR and RIP-qPCR were used to assess NCOA4 m^6^A methylation and YTHDF2 binding. A PE mouse model was established to assess in vivo effects and the potential rescue by Ferrostatin-1 (Fer-1).

**Results:**

WTAP expression and global m^6^A levels were reduced in PE placentas, accompanied by villous structural damage. Functionally, WTAP knockdown suppressed trophoblast proliferation and migration, induced G1 arrest, and enhanced oxidative stress, while WTAP overexpression had opposite effects. Mechanistically, WTAP promoted m^6^A methylation of NCOA4 mRNA and its YTHDF2-dependent degradation. In PE placentas, YTHDF2 was downregulated and NCOA4 upregulated, consistent with in vitro findings. NCOA4 overexpression impaired trophoblast function and increased ferroptosis, whereas silencing had protective effects. YTHDF2 knockdown and NCOA4 overexpression acted synergistically to exacerbate ferroptosis, both in trophoblasts and in a PE mouse model, leading to aggravated hypertension, proteinuria, and fetal growth restriction, which were partially reversed by Fer-1.

**Conclusion:**

WTAP suppresses ferroptosis in PE by enhancing YTHDF2-dependent m^6^A methylation and degradation of NCOA4. Disruption of this pathway exacerbates oxidative stress, trophoblast dysfunction, and adverse pregnancy outcomes.

**Supplementary Information:**

The online version contains supplementary material available at 10.1186/s13148-025-02004-w.

## Introduction

Preeclampsia (PE) is a severe hypertensive disorder during pregnancy that poses a significant threat to both maternal and neonatal health, and its pathogenesis remains complex and has not been fully elucidated [1, 2]. Ferroptosis is a recently identified form of regulated cell death that differs from classical forms of cell death. Its distinctive features include Fe-dependent accumulation of ROS and lipid peroxidation [3, 4]. It is implicated in a variety of pathological conditions, including cancer, ischemia–reperfusion injury, and neurodegenerative diseases [5]. The hallmark features of PE include placental insufficiency, endothelial dysfunction, and elevated oxidative stress, which together pose a serious risk to both maternal and fetal health [6].

Recent studies have revealed that oxidative stress and dysregulation of iron homeostasis play critical roles in PE [7]. Mechanisms associated with ferroptosis, such as the accumulation of reactive oxygen species (ROS) and the disruption of intracellular iron regulation, may directly contribute to placental dysfunction and maternal complications [8–10]. Despite the potential involvement of ferroptosis in PE, research in this area remains limited, and the precise molecular mechanisms underlying its contribution to PE pathogenesis require further in-depth investigation [11]. m^6^A RNA methylation is a common epigenetic modification that plays a key role in regulating biological processes such as cell proliferation, differentiation, and death [12]. WT1 associated protein (WTAP), an important component of the m^6^A methyltransferase complex, accurately regulates RNA m^6^A methylation levels [13]. Studies suggest that WTAP may influence cellular functions and pathological processes by regulating the m^6^A methylation of specific genes [13].

YTH N6-Methyladenosine RNA Binding Protein F2 (YTHDF2), a key m^6^A ‘reader’ protein, mediates mRNA stability, translation, and degradation by recognizing m^6^A sites [14]. Under cellular stress and disease progression, YTHDF2 precisely regulates metabolic and functional processes by targeting the mRNA stability of critical genes [15]. Although the role of YTHDF2 in ferroptosis and placental dysfunction has not been fully elucidated, it likely contributes to these processes by regulating the expression of ferroptosis-related genes [16]. Nuclear receptor coactivator 4 (NCOA4), a pivotal regulator of ferroptosis, mediates ferritin degradation and promotes iron release [17]. Aberrant NCOA4 expression may exacerbate oxidative stress and iron metabolism disorders, but its specific involvement in PE pathogenesis has not been thoroughly explored.

This study aims to integrate m^6^A methylation with ferroptosis-related mechanisms to investigate how WTAP and YTHDF2 regulate m^6^A modification of NCOA4, thereby influencing trophoblast function and elucidating ferroptosis-related mechanisms in PE. These findings may provide new insights into the pathogenesis of preeclampsia.

## Materials and methods

### Cell culture

The human extravillous trophoblast cell line HTR-8/SVneo (obtained from Wuhan Pricella Biotechnology Co., Ltd., China) was cultured in RPMI-1640 medium with 10% FBS and 1% penicillin–streptomycin (Meilunbio, China). Cells were incubated at 37 °C with 5% CO_2_. When the cell confluence reached approximately 80%, cells were passaged using 0.25% trypsin–EDTA solution (Beyotime, China). Some groups of cells were treated with ferrostatin-1 (Fer-1, 5 μM; MCE, USA) for 24 h [18].

### Human placental specimen collection

Placental tissues were obtained from normotensive pregnant women (n = 20) and patients with preeclampsia (n = 20) at the Affiliated Hospital of Qingdao University. The groups were matched for maternal age, gestational age, and relevant clinical parameters. Preeclampsia was diagnosed based on gestational age ≥ 20 weeks, blood pressure ≥ 140/90 mmHg on two occasions at least 4 h apart, and either new-onset proteinuria (protein/creatinine ratio ≥ 30 mg/mmol or 24 h protein ≥ 300 mg) or evidence of maternal organ or placental dysfunction. Fresh tissues were snap-frozen in liquid nitrogen or fixed in 4% paraformaldehyde (PFA) for histological analysis. All specimens were confirmed by pathologists. This study was approved by the Ethics Committee of the Affiliated Hospital of Qingdao University (Approval No. QYFYWZLL28706) and conducted in accordance with the Declaration of Helsinki.

### Animals

Seven-week-old unmated female and adult male C57BL/6J mice were purchased from Xingkang (China). Female mice were housed under specific-pathogen-free conditions with a 12 h light/dark cycle, temperature 22 ± 2 °C, humidity 50 ± 10%, and ad libitum access to food and water. Vaginal smears were performed to determine the estrous cycle; females in proestrus were paired with males at a 2:1 ratio. The day of vaginal plug detection was designated as gestational day (GD) 0. Pregnant mice were randomly assigned to experimental groups. To induce a preeclampsia-like phenotype, mice received subcutaneous injections of L-NAME (50 mg/kg/day, MedChemExpress, USA) from GD 7 to GD 18 [19]. For in vivo gene silencing, si-YTHDF2 or si-NCOA4 (Genechem, China) or negative control siRNA were administered via tail-vein injection on GD7. NCOA4 overexpression was achieved by tail-vein injection of AAV-CMV-NCOA4-OE (Genechem, China); control mice received equal volumes of AAV-empty vector. Following gene manipulation, mice were treated intraperitoneally with Fer-1 (2 μmol/kg) [18] or vehicle every other day from GD7 to GD17. Systolic blood pressure was measured non-invasively using tail-cuff plethysmography (IITC, USA) daily from GD7 to GD18. Mice were acclimated to the device for 3–5 days prior to measurements; at each time point at least three consecutive stable readings were recorded and averaged. Animals were monitored daily for health status and signs of distress throughout the study.

### Cell transfection

siRNA targeting YTHDF2 or NCOA4 and negative control siRNA (Genechem, China) were transfected into HTR-8/SVneo cells using Lipofectamine 2000 (Thermo Fisher, USA) according to the manufacturer's instructions. siRNA and Lipofectamine 2000 were diluted separately in 50 μL Opti-MEM, mixed, and incubated for 20 min at room temperature to form transfection complexes. After 6 h, the medium was replaced with RPMI-1640 medium with 10% FBS. Cells were harvested 24 h post-transfection for further analysis.

### CCK-8 assay

HTR-8/SVneo cells were seeded in 96-well plates and transfected with siRNA targeting NCOA4. After 48 h, CCK-8 solution (Beyotime, China) was added to each well, and the cells were incubated at 37 °C for 4 h. Absorbance was measured at 450 nm using a microplate reader (Thermo Fisher, USA).

### Transwell assay

Cells (1 × 10^5^ cells/mL) were suspended in 200 μL and added to the upper chamber, while 600 μL of complete medium was added to the lower chamber. After incubation for 48 h, the upper chamber was rinsed, and cells were fixed with 4% paraformaldehyde and stained with crystal violet for 10 min. The cells were examined under a microscope (Olympus, Japan).

### ROS assay

Cells were resuspended in serum-free medium and incubated with the ROS probe (Meilunbio, China) at 37 °C for 60 min. The intracellular ROS levels were detected using flow cytometry (Agilent Bio, USA). DCFH-DA probe was used, with excitation/emission wavelengths of 488/530 nm. The fluorescence intensity was recorded and analyzed.

### H&E staining

Placental tissues were fixed in 4% paraformaldehyde 24 h (Beyotime, China), dehydrated using a graded ethanol series, and cleared with xylene. Paraffin-embedded tissues were sectioned at a thickness of 4 μm. The sections were stained with hematoxylin and eosin (H&E) following routine protocols. Following staining, the slices were dehydrated using increasing ethanol concentrations, cleaned in xylene, and visualized under a light microscope (Olympus, Japan).

### Immunohistochemistry

The paraffin sections were heated in sodium citrate buffer at 95 °C for 10 min, followed by cooling to room temperature. Endogenous peroxidase was blocked with 3% hydrogen peroxide for 5 min, and non-specific binding was prevented by 5% BSA. The sections were incubated overnight at 4 °C with primary antibodies against WTAP (1:200; Proteintech, China) and YTHDF2 (1:300; ABclonal, China), followed by incubation with the appropriate secondary antibodies (1:2000; ZSGB, China). IHC was visualized with DAB and counterstained with hematoxylin.

### Wound healing assay

Cells were seeded in 6-well plates at a density of 5 × 10^5^ cells/well and cultured until reaching 90% confluence. Linear wounds were scratched with a sterile 200 μL pipette tip. After washing three times with PBS to remove cell debris, the cells were incubated in serum-free medium. Images of the wound area were captured at 0 h and 24 h using a phase-contrast microscope (Olympus, Japan). The wound closure rate was calculated using Image J software.

### Western blot

Total protein was extracted from tissues or cultured cells using RIPA lysis buffer (Solarbio, China) supplemented with 1% protease and phosphatase inhibitor cocktail (Beyotime, China). After centrifugation at 12,000×*g* for 15 min at 4 °C, supernatants were collected, and protein concentrations were quantified using a BCA Protein Assay Kit (Beyotime, China). Equal amounts of protein (20–30 μg) were resolved by SDS-PAGE and electrotransferred onto PVDF membranes (Millipore, USA). After blocking with 5% non-fat milk in TBST for 1 h at room temperature, membranes were incubated overnight at 4 °C with primary antibodies against WTAP (1: 1000; Proteintech, China), YTHDF2 (1: 10,000; ABclonal, China), NCOA4 (1: 1000; ABclonal, China), NRF2 (1: 2000; Abcam, UK), GPX4 (1: 1000; Proteintech, China), and GAPDH (1: 10,000; Proteintech, China) followed by incubation with HRP-conjugated secondary antibodies (1: 20,000; ZSGB, China) for 1 h at room temperature. Signals were detected using ECL reagents (Millipore, USA). β-actin was used as the internal loading control.

### Flow cytometry

Cells were rinsed with a pre-cooled D-Hanks solution at 4 °C, and fixed in 75% ethanol for 1 h. The fixative was removed by centrifugation, and the cells were rinsed again with D-Hanks solution. For each experimental group, three replicate wells were created. The staining solution was created by combining 40 × PI master mix, 100 × RNase A master mix, and 1 × D-Hanks at 25:10:1000 ratio.The solution was added to the cells, which were analyzed by flow cytometer (Beckman Coulter, USA) with 10,000 events recorded. Data were processed using FlowJo software (v10.8).

### Detection of Fe^2+^ levels

Fe^2^⁺ levels were measured using the CheKine™ Micro Ferrous Ion Content Assay Kit (Solarbio, China), cells or tissue homogenate were prepared and treated as directed. Absorbance at 593 nm was measured using a microplate reader (Thermo Fisher Scientific, USA). A standard curve was generated from the standards, and Fe^2^⁺ concentration was calculated from the absorbance values.

### Detection of MDA, GSH, GSSG, and urinary protein levels

The standard samples and test samples were diluted according to the instructions provided in the kit manual (Fankel, China). A microplate containing blank, standard, and sample wells was used. Standard solution was added to the standard wells, while diluent and sample were added to the sample wells. The plate was incubated at 37 °C for 30 min. After incubation, the wells were washed five times with wash buffer. Subsequently, enzyme-labeled reagent was added to each well, except the blank wells. The plate was incubated again at 37 °C for 30 min and washed as described above. Color developers A and B were then added, and the plate was incubated in the dark at 37 °C for 10 min. Finally, stop solution was added to each well. The absorbance was measured at 450 nm, with blank wells used for baseline correction.

### Analysis of m^6^A content

Total RNA was extracted using Trizol reagent and fragmented into approximately 100 nucleotides. The global m^6^A methylation level was quantified using the m^6^A RNA Methylation Kit (Fankel, China) according to the manufacturer's instructions. The absorbance at 450 nm was measured and normalized to the input RNA levels.

### Detection of IL-6

Serum IL-6 concentrations were determined using the Human IL-6 (Interleukin 6) ELISA Kit (Elabscience, China). The detection range of the kit was 10–500 pg/mL. Samples were diluted at a ratio of 1:10 with assay buffer, and absorbance was measured at 450 nm.

### qRT-PCR

Total RNA was extracted using TRIzol Universal reagent (TIANGEN, China), treated with DNase I (Thermo Fisher Scientific, USA), and quantified by NanoDrop™ Spectrophotometer (Thermo Fisher Scientific, USA). RNA was reversely transcribed into cDNA using the Hifair IIII 1 st Strand cDNA Synthesis SuperMix (YEASEN, China). The qPCR reaction system was prepared using the Hieff Fast Cell Direct Probe qRT-PCR Kit (YEASEN, China), and reactions were performed using a CFX96™ Real-Time PCR System (Bio-Rad, USA). Ct values were recorded and analyzed via the CT (2^−ΔΔCT^) method for subsequent data interpretation. The primers used in this study were listed in Table 1.

**Table 1:** Sequences of primers used for qRT-PCR

Gene	Forward primer (5′–3′)	Reverse primer (5′–3′)
Human		
WTAP	CTTCCCAAGAAGGTTCGATTGA	TCAGACTCTCTTAGGCCAGTTAC
YTHDF2	AGCCCCACTTCCTACCAGATG	TGAGAACTGTTATTTCCCCATGC
GPX4	GAGGCAAGACCGAAGTAAACTAC	CCGAACTGGTTACACGGGAA
NRF2	AACTTTCGGAATTATTGGCAAGC	CGTCTCTGGTCAGATTTGACAGT
NCOA4	CCTTCCAAGACCAGAGTGGC	TCTCCAGGAAGGGCCCAATA
GAPDH	GCTCTCTGCTCCTCCTGTTC	GCAGGAGGCATTGCTGATGA
Mouse		
Nrf2	GGTCACGCTAATGCAGACAAT	TCTTCTCAGGGGTATTCGCTTT
Gpx4	GATGGAGCCCATTCCTGAACC	CCCTGTACTTATCCAGGCAGA
GAPDH	TGGATTTGGACGCATTGGTC	TTTGCACTGGTACGTGTTGAT

### RNA-Seq

Total RNA was extracted using Trizol reagent and its concentration was determined using a NanoDrop™ 2000 spectrophotometer (Thermo Fisher, USA) and Agilent 2100 Bioanalyzer (RNA Integrity Number, RIN > 8.0). RNA samples that met quality standards were used for mRNA enrichment, followed by quality control, cDNA library building, and additional quality assessment. Professional service providers then did high-throughput sequencing. Raw sequencing data were processed with SOAPnuke software to remove low-quality reads and adaptors. Data were normalized using DESeq2 (v1.38.3), and differentially expressed genes (DEGs) were identified with *|log2(fold change)|*> 1 and* q* < 0.05, where q represents the adjusted p-value after Benjamini Hochberg correction to control the false discovery rate (FDR), which were then displayed using volcano plots. DEGs were visualized using volcano plots. Functional annotation was performed via Gene Ontology (GO) and Kyoto Encyclopedia of Genes and Genomes (KEGG) enrichment analysis.

### RNA immunoprecipitation followed by qPCR (RIP-qPCR)

RIP was performed using the RIP Assay Kit (Beyotime, China) according to the manufacturer’s instructions. Briefly, cells were lysed in RIP lysis buffer containing RNase inhibitor. The lysates were incubated overnight at 4 °C with magnetic beads pre-coupled with anti-YTHDF2 antibody or IgG control. After extensive washing, RNA–protein complexes were eluted, and RNA was extracted and purified. The purified RNA was reverse-transcribed into cDNA, and the enrichment of NCOA4 mRNA fragments was quantified by qRT-PCR.

### m^6^A RNA immunoprecipitation (MeRIP-qPCR)

MeRIP-qPCR was performed using m^6^A RNA Immunoprecipitation Kit (Bersinbio, China) according to the manufacturer’s instructions. Total RNA was fragmented and incubated with anti-m^6^A antibody in IP buffer at 4 °C for 4 h. Protein A/G magnetic beads were prepared and added for an additional 1-h incubation. After washing, m^6^A-modified RNA was eluted, digested with proteinase K, and purified. Enriched RNA and input samples were reverse-transcribed and analyzed by qRT-PCR.

### Actinomycin D

To assess mRNA stability, transfected cells were treated with Actinomycin D (5 µg/mL; MCE, USA) to inhibit transcription. Cells were harvested at 0, 1, 2, 4, and 6 h post-treatment, and total RNA was extracted. NCOA4 mRNA levels were quantified by qRT-PCR.

### Statistical analysis

Statistical analyses were performed with GraphPad Prism 10.0. Data are mean ± SD of three independent experiments. t-tests were used for two-group comparisons. All multiple-group comparisons used one-way ANOVA, followed by Tukey’s post hoc test for pairwise comparisons. *P* < 0.05 was considered significant.

## Results

### WTAP expression and m^6^A methylation levels are reduced in PE placentas

**Fig. 1:**
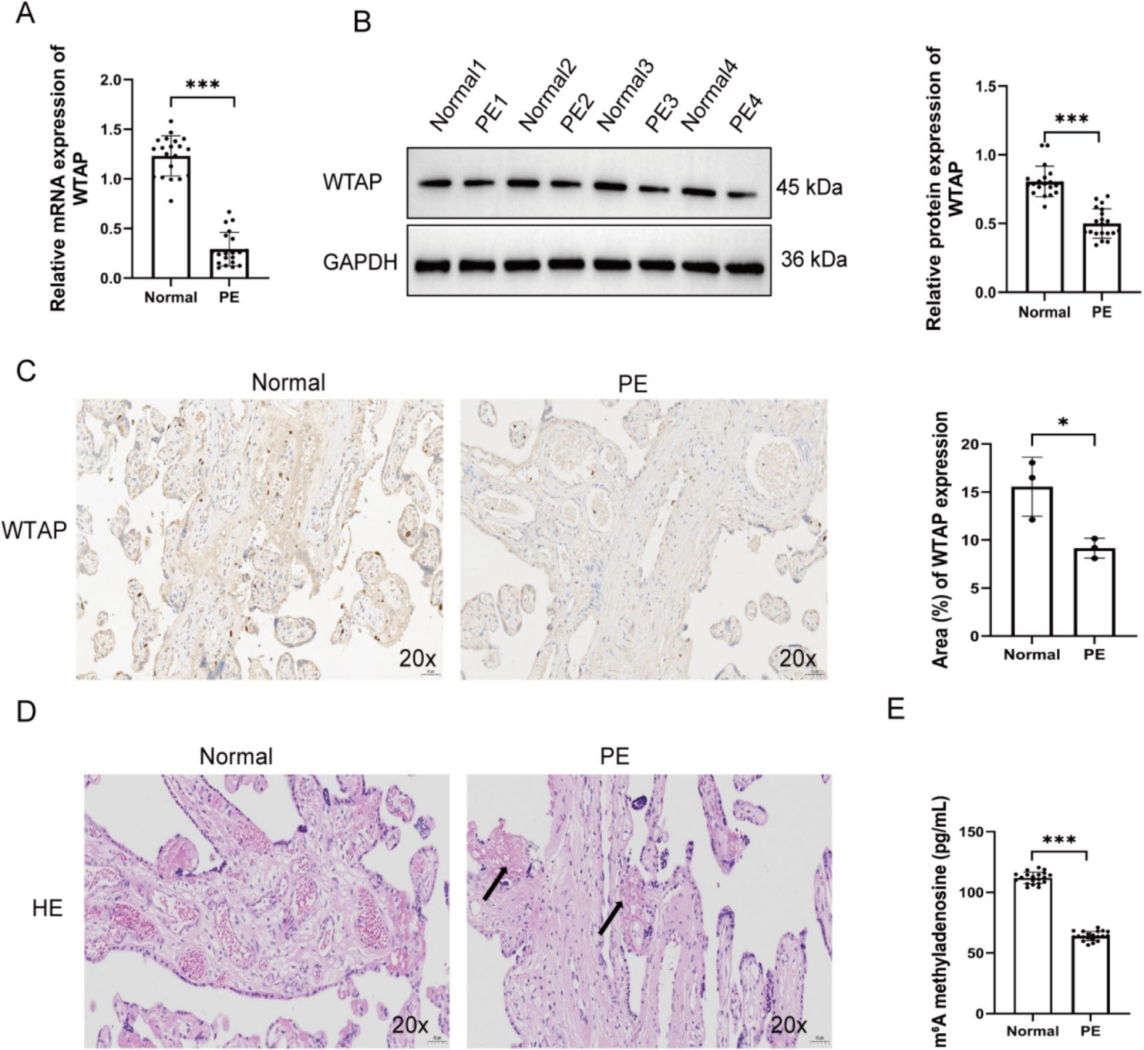
WTAP expression and m^6^A methylation levels are reduced in PE placentas. WTAP expression in PE placentas was assessed by qRT-PCR (**A**), Western blot (**B**), and IHC (20×) (**C**). **D** HE staining of PE placental tissue (20×). **E** m^6^A methylation levels in PE placentas, *P < 0.05, ***P < 0.001

We initially investigated the expression of WTAP in placental tissues from healthy and preeclamptic pregnancies. Compared to the control group, WTAP expression was significantly downregulated in PE placentas at both the mRNA (Fig. 1A) and protein (Fig. 1B) levels. Immunohistochemical staining demonstrated a markedly reduced WTAP-positive area in placental tissues from the PE group (Fig. 1C). Furthermore, histopathological analysis revealed significant histopathological abnormalities in PE placentas, including villous structural disruption, stromal edema, disordered trophoblasts, and reduced vascular density, which are indicative of ischemic injury (Fig. 1D). In line with the reduced WTAP expression, m^6^A methylation levels were also found to be significantly decreased in the PE group (Fig. 1E). Collectively, these findings indicate that reduced WTAP expression and m^6^A hypomethylation are associated with the pathological features of PE placentas.

### WTAP promotes trophoblast proliferation, migration, and redox balance

**Fig. 2:**
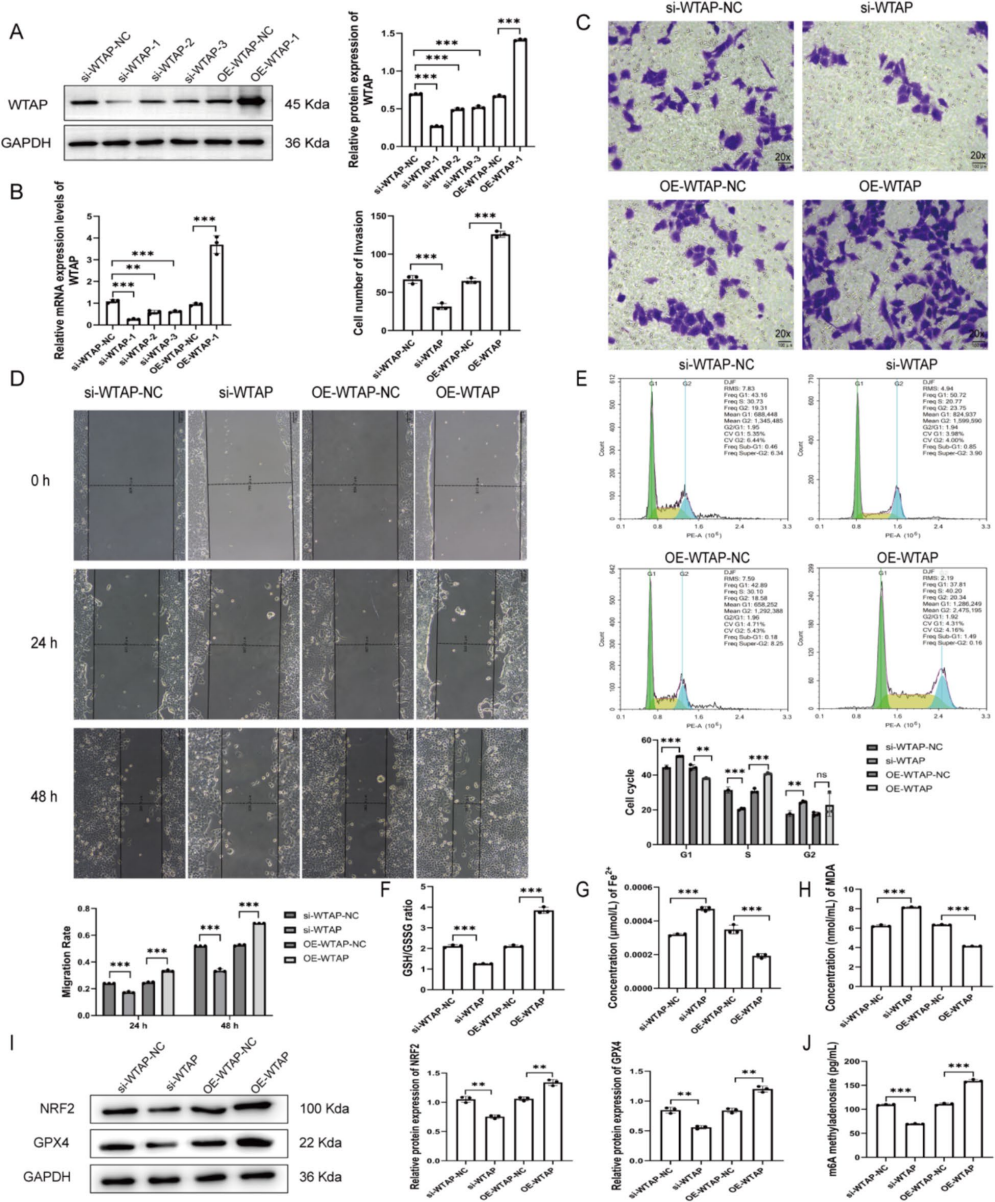
WTAP promotes trophoblast proliferation, migration, and redox balance. WTAP transfection efficiency assessed by qRT-PCR (**A**) and Western blot (**B**). Cell invasion assessed by Transwell assay (20×) (**C**). Cell migration assessed by wound healing assay (**D**). Cell cycle distribution analyzed by flow cytometry (**E**). GSH/GSSG ratio (**F**). Intracellular Fe^2^⁺ and MDA levels (**G**–**H**). NRF2 and GPX4 protein expression by Western blot (**I**) and mRNA expression by qRT-PCR (**J**), *P < 0.05, **P < 0.01, ***P < 0.001, *ns P* > 0.05

Given that WTAP and global m^6^A levels were reduced in PE placentas, we next examined the functional consequences of altered WTAP in HTR-8/SVneo cells. WTAP was silenced using siRNAs (si-WTAP) or overexpressed via plasmid transfection (OE-WTAP); transfection efficiency was confirmed by qRT-PCR and western blot (Fig. 2A, B). WTAP knockdown significantly inhibited trophoblast migration and invasion, whereas WTAP overexpression enhanced these processes (Fig. 2C, D). Flow cytometric analysis showed that si-WTAP induced G1 arrest, while OE-WTAP facilitated the G1/S transition (Fig. 2E). Regarding redox homeostasis, OE-WTAP increased the GSH/GSSG ratio and reduced intracellular Fe^2^⁺ and MDA levels (Fig. 2F–H), accompanied by upregulation of the antioxidant proteins NRF2 and GPX4 (Fig. 2I). Consistent with the tissue data, WTAP knockdown decreased global m^6^A levels in HTR-8/SVneo cells (Fig. 2J). Collectively, in HTR-8/SVneo cells, WTAP overexpression promoted migration and cell-cycle progression, accompanied by improved redox status.

### WTAP knockdown reduces YTHDF2 and increases NCOA4 expression, consistent with PE placentas

**Fig. 3:**
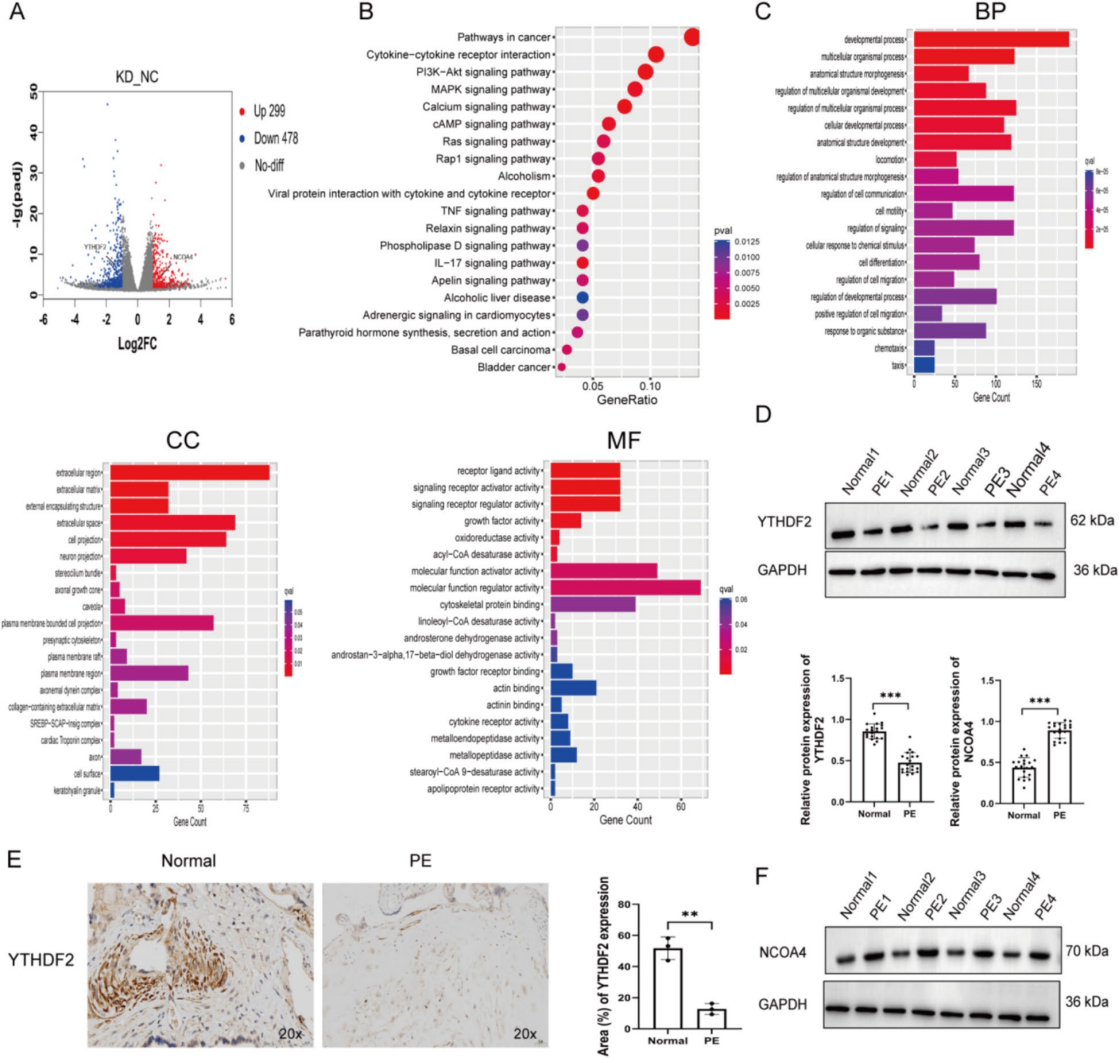
WTAP knockdown reduces YTHDF2 and increases NCOA4 expression, consistent with PE placentas. **A** Volcano plot of DEGs after WTAP knockdown (A). KEGG pathway enrichment of DEGs (**B**). GO enrichment of DEGs (**C**). YTHDF2 protein expression in placentas by Western blot (**D**) and IHC (20×) (**E**). NCOA4 protein expression in placentas by Western blot, **P < 0.01, ***P < 0.001

To investigate mechanisms downstream of WTAP, we performed RNA-seq in HTR-8/SVneo cells following WTAP knockdown. Differential expression analysis identified 777 DEGs, including 299 upregulated and 478 downregulated genes (Fig. 3A). KEGG enrichment revealed significant enrichment of the PI3K–Akt, MAPK, TNF, and cAMP signaling pathways (Fig. 3B), which are implicated in proliferation, motility, and oxidative-stress responses. GO analysis showed enrichment for biological processes related to cell migration, cell motility, and chemotaxis (Fig. 3C). Notably, the m^6^A reader YTHDF2 was significantly downregulated, whereas NCOA4 was upregulated upon WTAP knockdown (Supplementary Table 1). We next assessed placental tissues and found that YTHDF2 protein was reduced in PE placentas compared with controls by western blot and immunohistochemistry (Fig. 3D, E), while NCOA4 protein was increased (Fig. 3F). In summary, WTAP knockdown was associated with transcriptional alterations in trophoblasts, characterized by reduced YTHDF2 and elevated NCOA4 expression, changes that were also observed in PE placentas.

### WTAP regulates NCOA4 expression and NCOA4 affects HTR-8/SVneo Cell Function

**Fig. 4:**
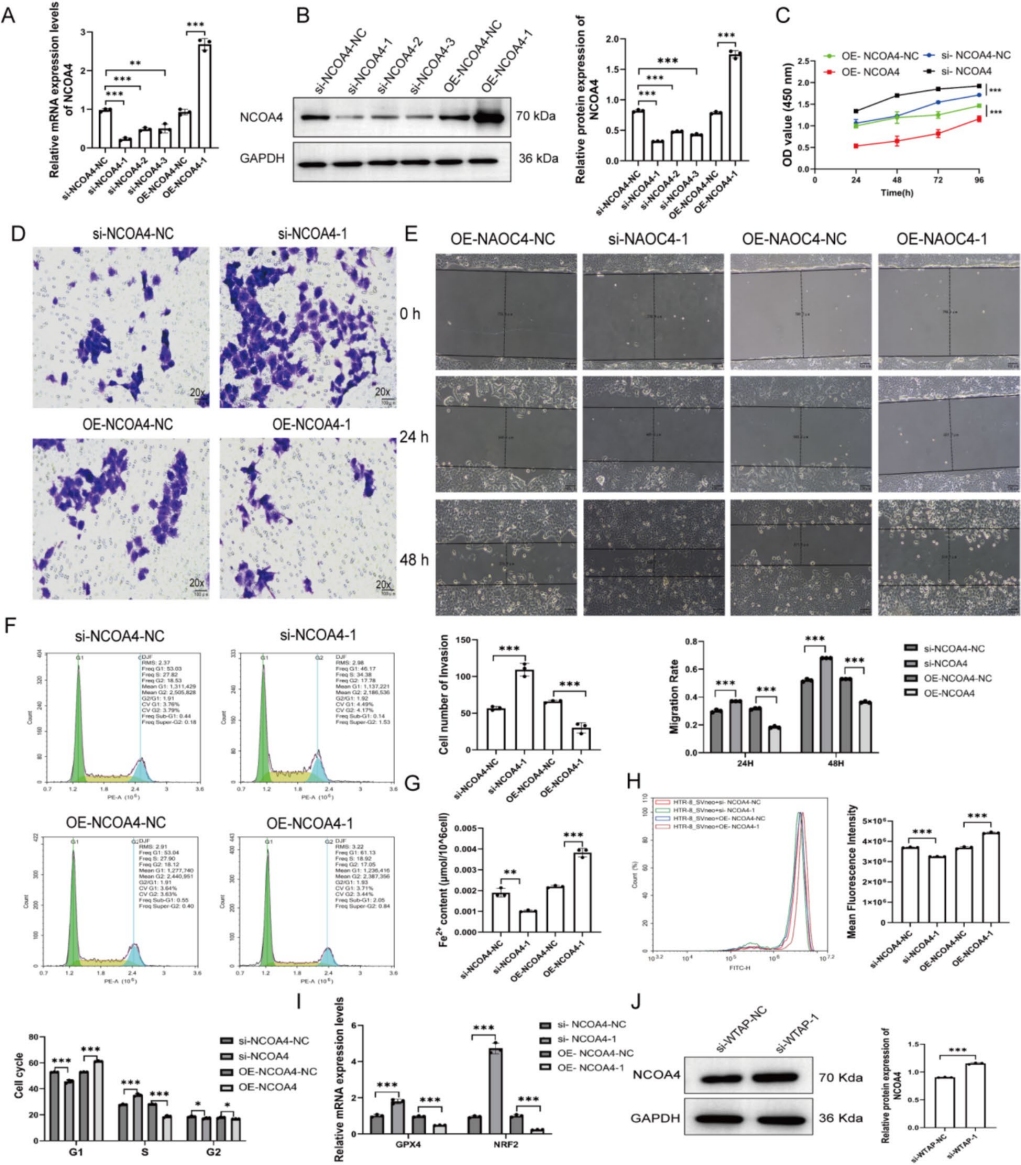
WTAP regulates NCOA4 expression and NCOA4 affects HTR-8/SVneo cell function. NCOA4 transfection efficiency assessed by qRT-PCR (**A**) and Western blot (**B**). Cell proliferation by CCK-8 assay (**C**). Cell invasion by Transwell assay (20×) (**D**). Cell migration by wound healing assay (**E**). Cell cycle distribution by flow cytometry (**F**). Intracellular Fe^2^⁺ levels (**G**). ROS levels by fluorescence intensity (**H**). NRF2 and GPX4 mRNA expression by qRT-PCR (**I**). NCOA4 protein expression by Western blot (**J**) The protein expression of NCOA4 by Western blot. *P < 0.05, **P < 0.01, ***P < 0.001

To investigate the role of NCOA4 in HTR-8/SVneo cells, we modulated its expression using siRNA (si-NCOA4) and overexpression (OE-NCOA4) (Fig. 4A, B). CCK-8 assays showed that si-NCOA4 significantly enhanced cell proliferation, whereas OE-NCOA4 inhibited it (Fig. 4C). OE-NCOA4 suppressed cell invasion and migration, while si-NCOA4 promoted these processes (Fig. 4D, E). Flow cytometry analysis revealed that OE-NCOA4 induced G1 phase arrest, whereas si-NCOA4 promoted G1/S transition (Fig. 4F). Moreover, OE-NCOA4 increased intracellular Fe^2^⁺ and ROS levels and decreased the expression of antioxidant proteins GPX4 and NRF2, whereas si-NCOA4 exerted opposite effects (Fig. 4G–I). Importantly, si-WTAP increased NCOA4 protein levels, indicating that WTAP negatively regulates NCOA4 expression (Fig. 4J). Collectively, these results suggest that NCOA4 negatively regulates trophoblast proliferation,migration, and redox balance.

### WTAP promotes YTHDF2-mediated m^6^A-dependent degradation of NCOA4

**Fig. 5:**
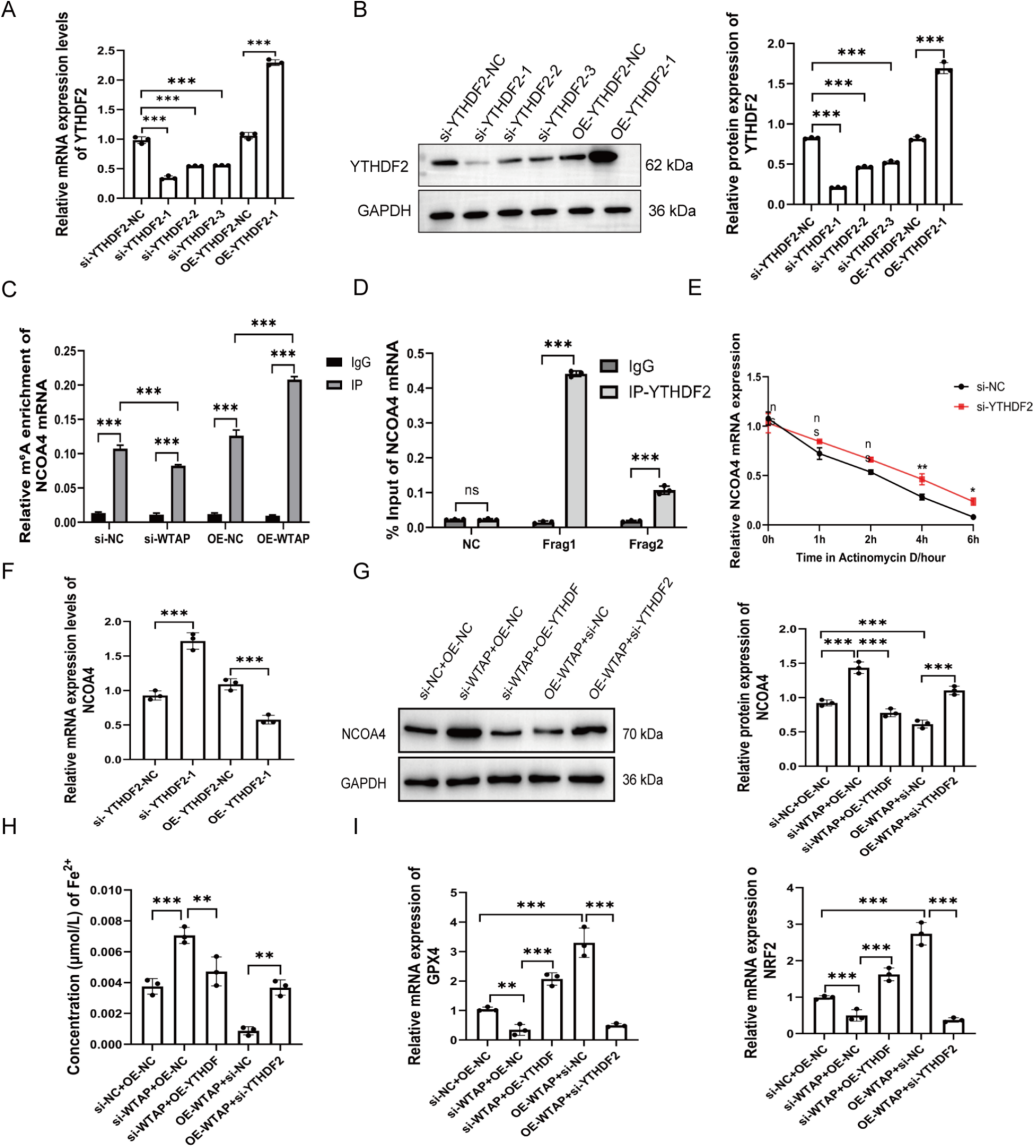
WTAP promotes YTHDF2-mediated m^6^A-dependent degradation of NCOA4. YTHDF2 transfection efficiency by qRT-PCR (**A**) and Western blot (**B**). (**C**) m^6^A enrichment on NCOA4 mRNA assessed by MeRIP-qPCR. (**D**) YTHDF2 binding to NCOA4 mRNA assessed by RIP-qPCR. (**E**) NCOA4 mRNA stability assessed by Actinomycin D assay. (**F**) NCOA4 mRNA expression by qRT-PCR. (**G**) NCOA4 protein expression by Western blot. (**H**) Intracellular Fe^2^⁺ concentrations. (**I**) NRF2 and GPX4 expression by qRT-PCR. **P < 0.01, ***P < 0.001

To investigate whether WTAP regulates NCOA4 via m^6^A modification, we first confirmed YTHDF2 transfection efficiency by qRT-PCR and Western blot (Fig. 5A, B). MeRIP-qPCR analysis showed that WTAP overexpression markedly increased m^6^A enrichment on NCOA4 mRNA, whereas WTAP knockdown reduced it (Fig. 5C). RIP-qPCR further demonstrated that YTHDF2 specifically binds NCOA4 mRNA at the predicted m^6^A sites (Fig. 5D). Functional assays revealed that YTHDF2 knockdown stabilized NCOA4 mRNA, as determined by Actinomycin D treatment (Fig. 5E), consistent with qRT-PCR results showing negative regulation of NCOA4 by YTHDF2 (Fig. 5F). We then examined downstream effects of WTAP/YTHDF2-mediated NCOA4 regulation. WTAP knockdown increased NCOA4 protein and intracellular Fe^2^⁺ levels while reducing GPX4 and NRF2, and these changes were partially reversed by YTHDF2 overexpression. Conversely, WTAP overexpression decreased NCOA4 and Fe^2^⁺ and upregulated GPX4 and NRF2, effects attenuated by YTHDF2 knockdown (Fig. 5G–I). Collectively, these results indicate that WTAP promotes YTHDF2-dependent m^6^A-mediated degradation of NCOA4, thereby modulating its expression and downstream redox homeostasis in HTR-8/SVneo cells.

### YTHDF2 downregulation and NCOA4 overexpression synergistically induce ferroptosis

**Fig. 6:**
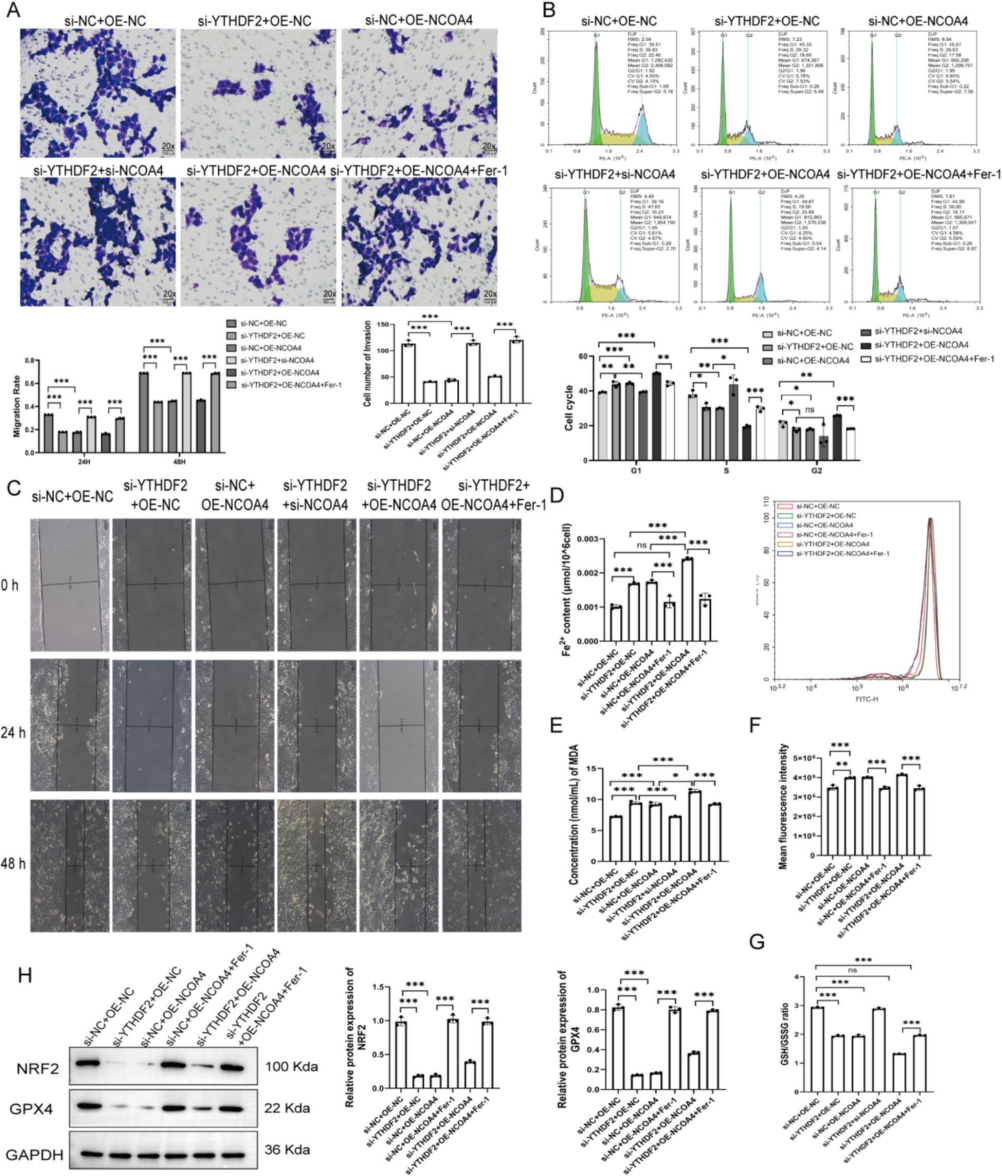
YTHDF2 downregulation and NCOA4 overexpression synergistically induce ferroptosis. **A** Cell invasion by Transwell assay (20 ×). **B** Cell cycle distribution by flow cytometry. **C** Cell migration by wound healing assay. **D**–**E** Intracellular Fe^2^⁺ and MDA levels. **F** ROS levels by fluorescence intensity. **G** GSH/GSSG ratio. **H** NRF2 and GPX4 protein expression by Western blot. *P < 0.05, **P < 0.01, ***P < 0.001, *n.s. P* > 0.05

To investigate the functional interaction between YTHDF2 and NCOA4, HTR-8/SVneo cells were transfected with si-YTHDF2 and/or OE-NCOA4. Both treatments individually suppressed cell invasion and migration, with the combined treatment showing the strongest effect. Co-treatment with the ferroptosis inhibitor Fer-1 partially rescued these effects (Fig. 6A, C). Similarly, si-YTHDF2 or OE-NCOA4 induced G1-phase arrest, which was mitigated by Fer-1 (Fig. 6B). Both treatments individually increased intracellular Fe^2^⁺ (Fig. 6D), MDA (Fig. 6E), and ROS levels (Fig. 6F). These effects were further amplified in co-treated cells; notably, Fer-1 treatment significantly attenuated these increases. Consistent with this, the intracellular GSH/GSSG ratio was reduced and the expression of NRF2 and GPX4 was downregulated following YTHDF2 knockdown or NCOA4 overexpression, whereas Fer-1 partially reversed these alterations (Fig. 6G, H). These findings indicate that YTHDF2 downregulation and NCOA4 overexpression synergistically exacerbate ferroptosis in trophoblasts.

### YTHDF2-mediated regulation of NCOA4 promotes ferroptosis in PE mice

**Fig. 7:**
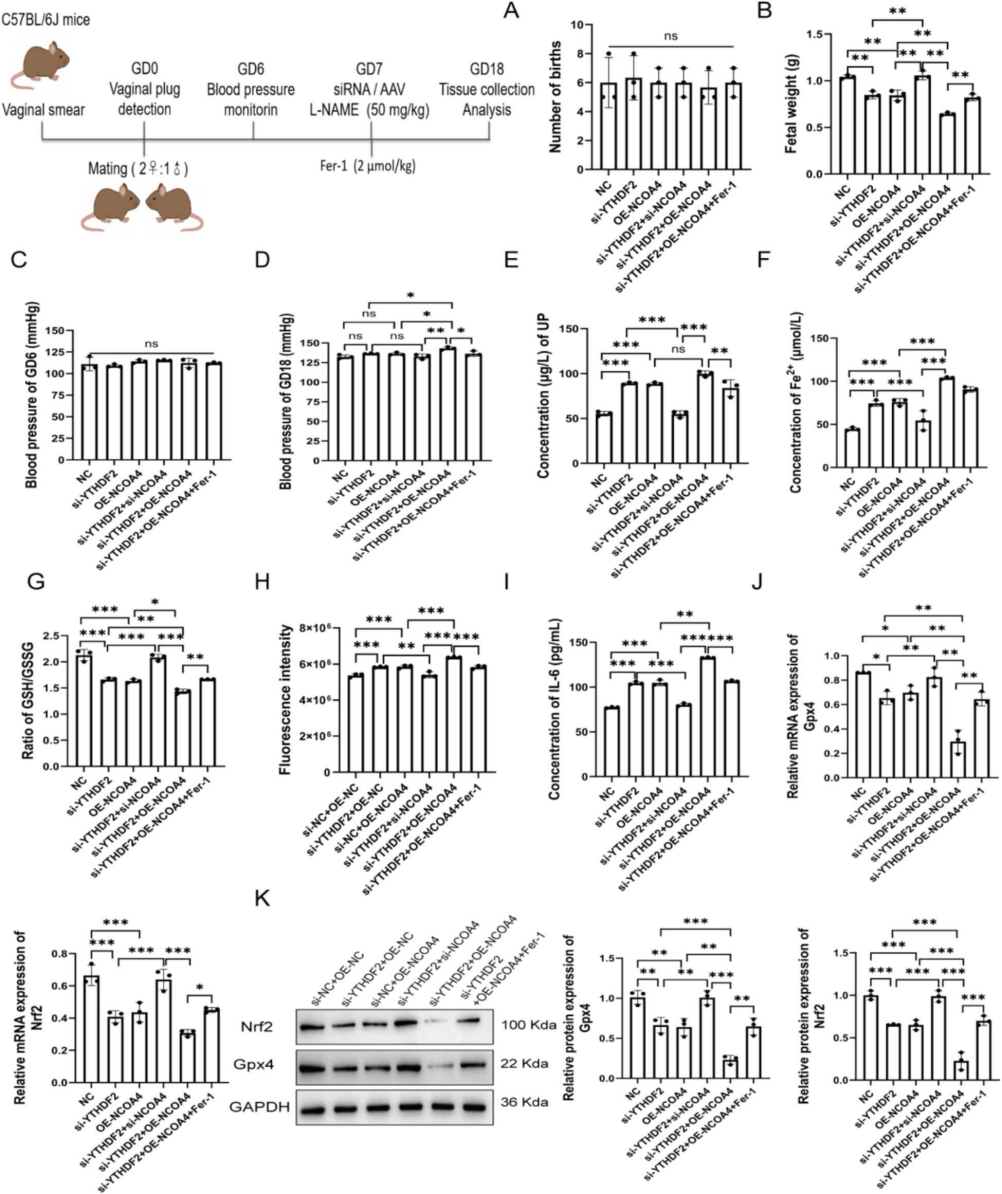
YTHDF2-mediated regulation of NCOA4 promotes ferroptosis in PE mice. **A** Number of births. **B** Fetal body weight. Systolic blood pressure at GD6 (**C**) and GD18 (**D**). **E** Urinary protein levels in mice. **F** Fe^2+^ concentrations in mice placental tissues. **G** GSH/GSSG ratio. **H** ROS levels by fluorescence intensity. **I** Serum IL-6 levels. **J**–**K** Nrf2 and Gpx4 expression by qRT-PCR and Western blot. *P < 0.05, *P < 0.05, **P < 0.01, ***P < 0.001, *n.s. P* > 0.05

We further evaluated the in vivo effects of YTHDF2 and NCOA4 modulation in a PE mouse model. Fer-1 treatment, alone or combined with YTHDF2 knockdown and NCOA4 overexpression, did not significantly affect live birth numbers (Fig. 7A). YTHDF2 knockdown or NCOA4 overexpression reduced fetal weight, with the combined treatment causing the greatest decrease; this effect was partially rescued by Fer-1 (Fig. 7B). Combined YTHDF2 knockdown and NCOA4 overexpression increased maternal systolic blood pressure in late gestation, while early gestation remained unaffected (Fig. 7C, D). Urinary protein levels were significantly elevated in mice with YTHDF2 knockdown or NCOA4 overexpression, and the combined treatment further increased proteinuria, which was partially reversed by Fer-1 administration (Fig. 7E). Biochemical assays of placental tissues showed elevated Fe^2^⁺, reduced GSH/GSSG ratio, increased ROS, and higher IL-6 levels, which were partially reversed by Fer-1 (Fig. 7F–I). Gpx4 and Nrf2 expression was suppressed (Fig. 7J, K). These results demonstrate that YTHDF2 downregulation and NCOA4 overexpression promote ferroptosis and impair fetal development in PE mice.

## Discussion

In this study, we investigated how WTAP regulates ferroptosis in trophoblasts through an m^6^A/YTHDF2–NCOA4 pathway and its contribution to the pathogenesis of PE. We found that WTAP expression was markedly downregulated in PE placentas, accompanied by a global reduction in m^6^A methylation. As an essential component of the m^6^A methyltransferase complex, WTAP plays a key role in post-transcriptional regulation by modulating m^6^A modification on target transcripts [20, 21]. This observation is in line with a previous report of WTAP downregulation in placental insufficiency [21], but our data further connect it to ferroptosis, a mechanism that has received little attention in PE pathogenesis.

Functionally, WTAP deficiency significantly impaired trophoblast proliferation, migration, and invasion, whereas WTAP overexpression promoted cell motility. These cellular changes were closely associated with redox imbalance: WTAP knockdown elevated intracellular Fe^2^⁺ and MDA levels while reducing antioxidant defenses such as GPX4 and NRF2, whereas WTAP overexpression increased the GSH/GSSG ratio and restored antioxidant protein expression [22, 23]. These results suggest that WTAP is essential for placental homeostasis, and loss of WTAP promotes oxidative stress and ferroptosis, thereby contributing to PE pathogenesis [24]. WTAP-mediated m^6^A regulation of ferroptosis has been documented in cancer biology [25, 26]. Our results extend this concept to placental pathology and support the possibility that WTAP may serve as a therapeutic target in PE [27]. Nevertheless, whether restoring WTAP expression alone is sufficient to reverse PE remains uncertain, as compensatory mechanisms in vivo cannot be excluded [28].

NCOA4, a pivotal regulator of ferroptosis, mediates ferritinophagy and releases free Fe^2^⁺, thereby driving oxidative stress [29, 30]. In our study, NCOA4 overexpression aggravated ferroptosis and suppressed trophoblast proliferation and migration, consistent with prior reports [31, 32]. NCOA4 has been implicated in ischemic injury [33]. WTAP deficiency markedly increased NCOA4 expression, suggesting that WTAP protects trophoblasts from ferroptosis at least partly by repressing NCOA4. Importantly, our mechanistic experiments showed that WTAP promotes m^6^A modification of NCOA4 mRNA, which facilitates YTHDF2-mediated recognition and degradation. In PE placentas, reduced WTAP expression was accompanied by decreased m^6^A enrichment on NCOA4 mRNA, impaired YTHDF2 binding, and consequent NCOA4 accumulation, thereby promoting ferroptosis. In addition, YTHDF2 knockdown stabilized NCOA4 mRNA, underscoring its central role in this regulatory axis [34]. The interplay between YTHDF2 and NCOA4 was further supported by functional assays. Either YTHDF2 downregulation or NCOA4 overexpression alone suppressed trophoblast invasion and migration, promoted G1-phase arrest, and increased Fe^2^⁺, ROS, and lipid peroxidation; combined treatment amplified these effects. Notably, the ferroptosis inhibitor Fer-1 partially rescued these alterations, indicating that the WTAP–YTHDF2–NCOA4 pathway primarily affects trophoblast function via ferroptosis.

In vivo, disruption of YTHDF2 or overexpression of NCOA4 significantly exacerbated oxidative stress and ferroptosis in the placenta, including elevated maternal blood pressure, proteinuria, and reduced fetal weight [35–37]. When both manipulations were applied simultaneously, the phenotype was most severe, consistent with their synergistic effects observed in vitro. Biochemical assays confirmed increased placental Fe^2^⁺, ROS, and IL-6, decreased GSH/GSSG ratios, and downregulation of Gpx4 and Nrf2. Treatment with Fer-1 attenuated these abnormalities, further supporting a central role for ferroptosis in PE pathology [38]. Future studies using genetic PE models may help strengthen the translational relevance of these findings [39].

In conclusion, WTAP serves as a central regulator of placental homeostasis by controlling NCOA4 mRNA degradation via YTHDF2-dependent m^6^A methylation. Loss of WTAP leads to NCOA4 accumulation, ferroptosis, and PE-like features, highlighting a critical molecular mechanism underlying PE pathogenesis. Our findings also raise the possibility that therapeutic targeting of the WTAP–YTHDF2–NCOA4 axis could mitigate ferroptosis and improve placental function. Our findings indicate that targeting this pathway, using either ferroptosis inhibitors or approaches that restore WTAP activity, may help protect placental function. Nevertheless, it is important to acknowledge the study’s limitations: most experiments were conducted in vitro or in limited animal models, and the effects of targeting this axis in humans remain unknown. Future work using genetic models of PE and clinically relevant interventions will be essential to determine the therapeutic potential and safety of this approach.

Overall, this study identifies WTAP as a key regulator of placental ferroptosis and establishes the WTAP–YTHDF2–NCOA4 pathway as a potential therapeutic target for preeclampsia.

## Supplementary Information

Additional file1

Additional file2

## Data Availability

The RNA-seq datasets supporting the conclusions of this article are available in the GEO repository, under Accession Number GSE302952.

## Abbreviations

PE: Preeclampsia
NCOA4: Nuclear receptor coactivator 4
NRF2: Nuclear factor erythroid 2-related factor 2
MDA: MalondialdehydeGPX4: Glutathione peroxidase 4
ROS: Reactive Oxygen Species
DEGs: Differentially expressed genes
GO: Gene Ontology
KEGG: Kyoto Encyclopedia of Genes and Genomes
