# Supplementary material for: WTAP regulates NCOA4-mediated ferroptosis via a YTHDF2-dependent mechanism in preeclampsia
Source: Clin Epigenetics. 2025 Nov 19;17:195. doi: 10.1186/s13148-025-02004-w (PMC12628531; doi:10.1186/s13148-025-02004-w)

Figure1B WTAP-1(as shown in this study)

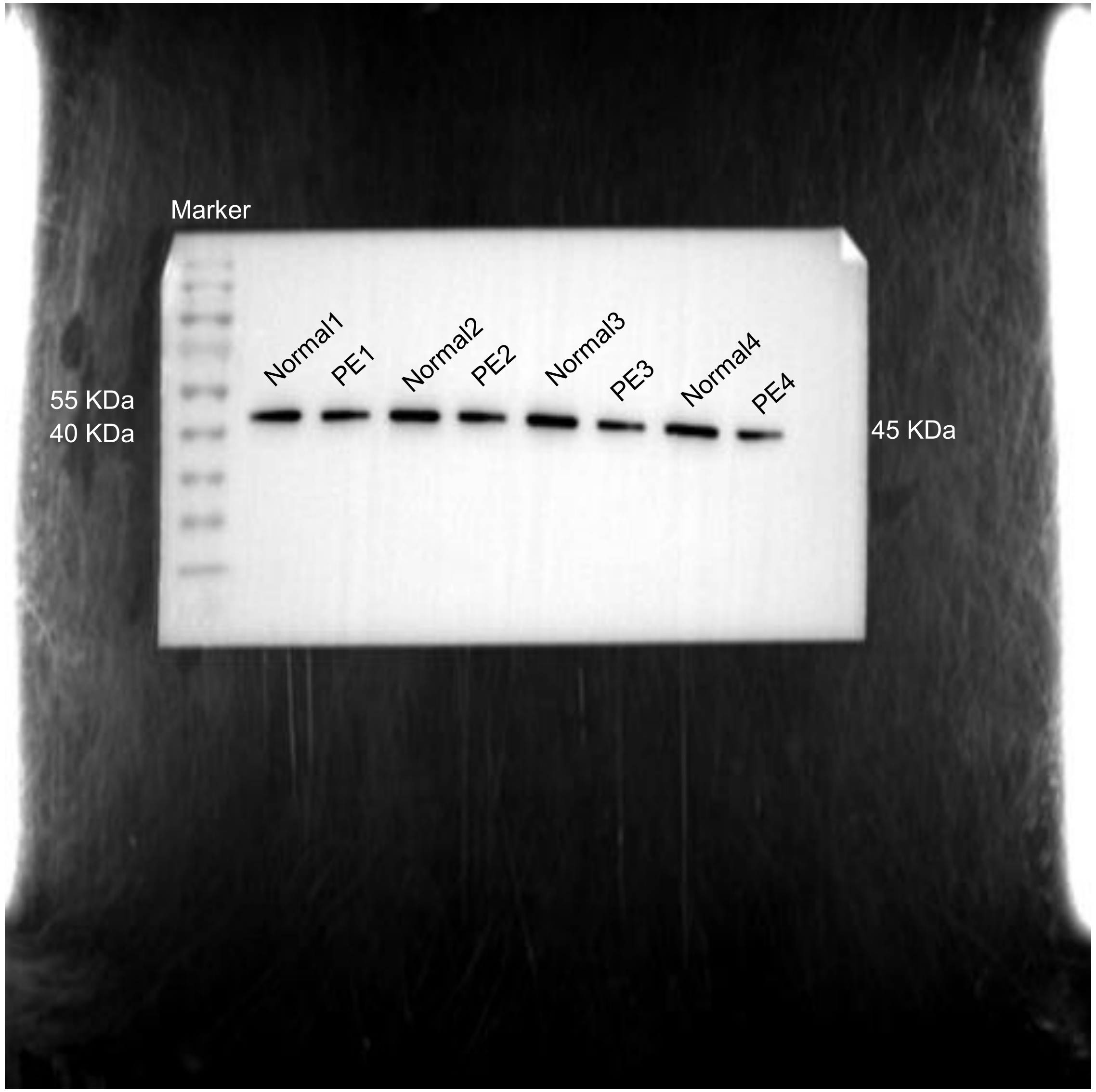

Figure1B WTAP-2

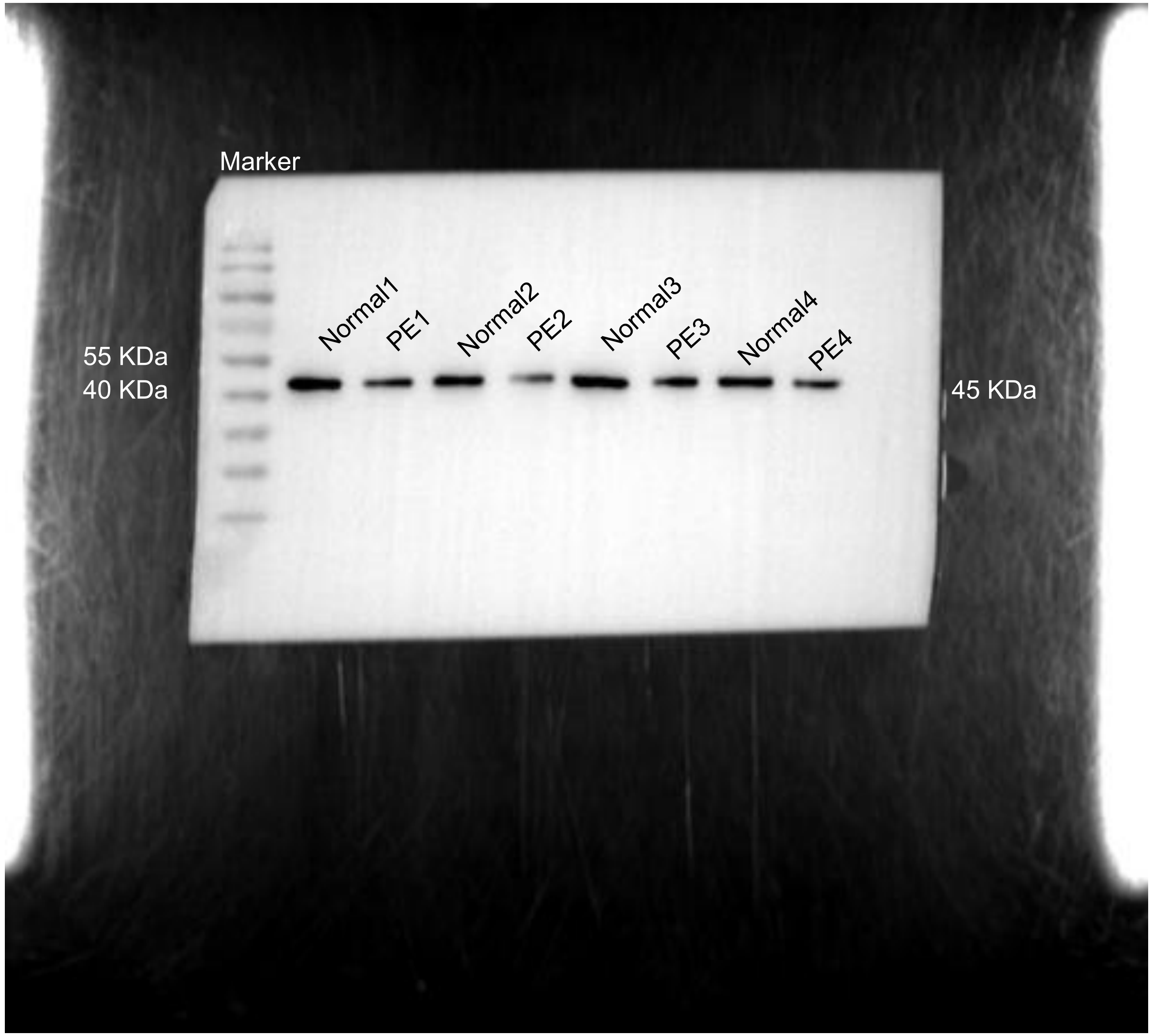

Figure1B WTAP-3

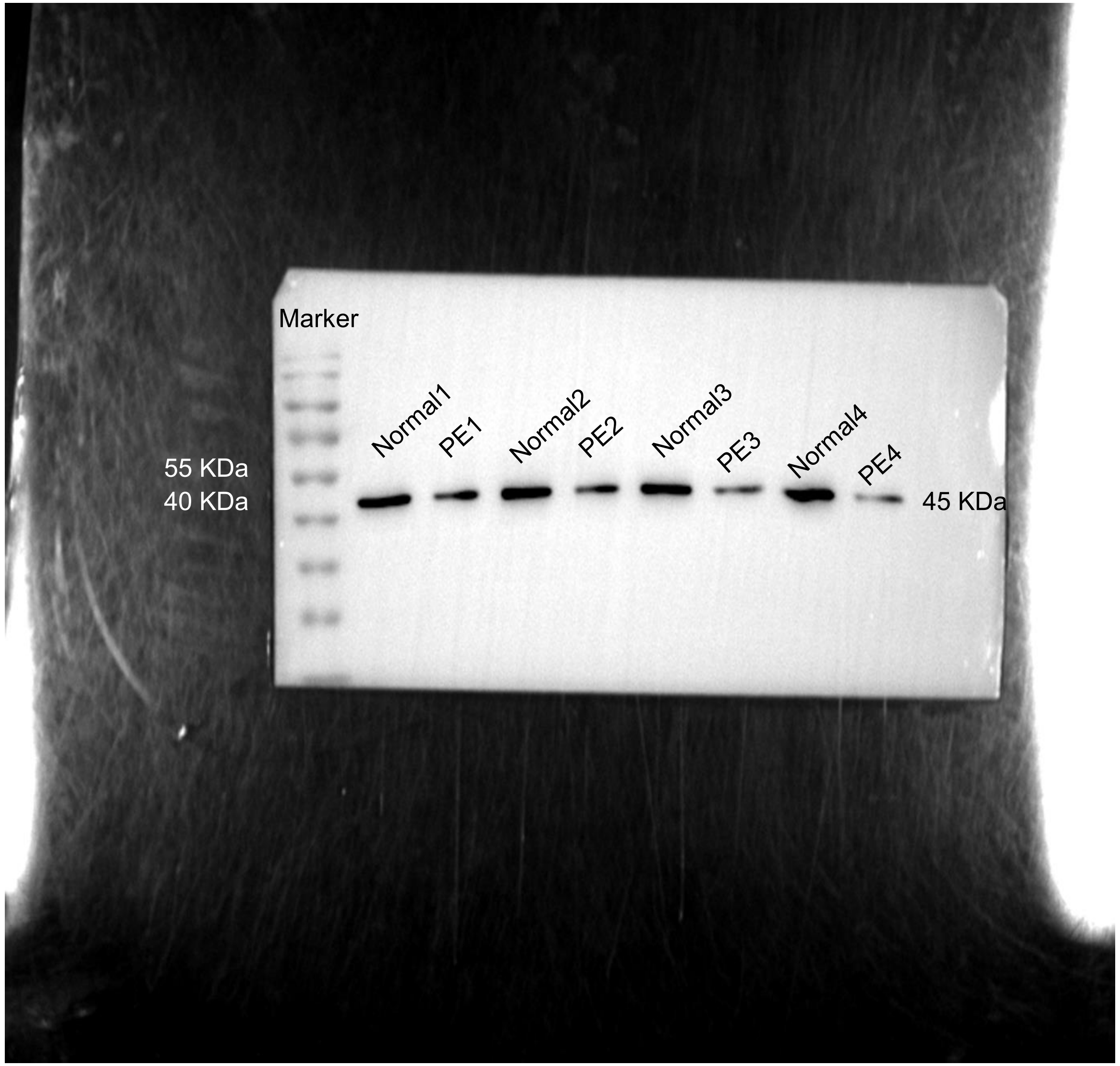

Figure1B GAPDH-1(as shown in this study)

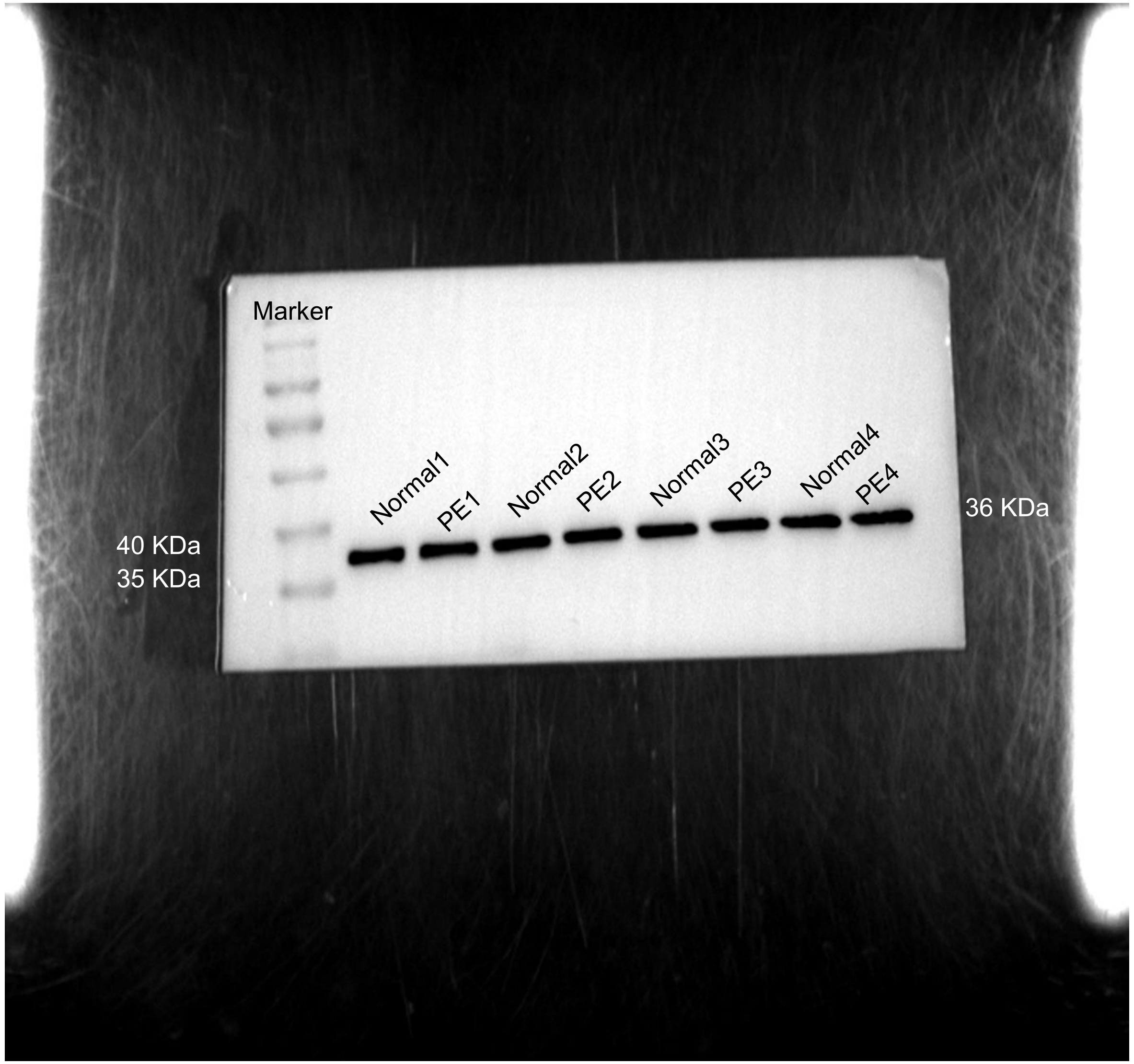

Figure1B GAPDH-2

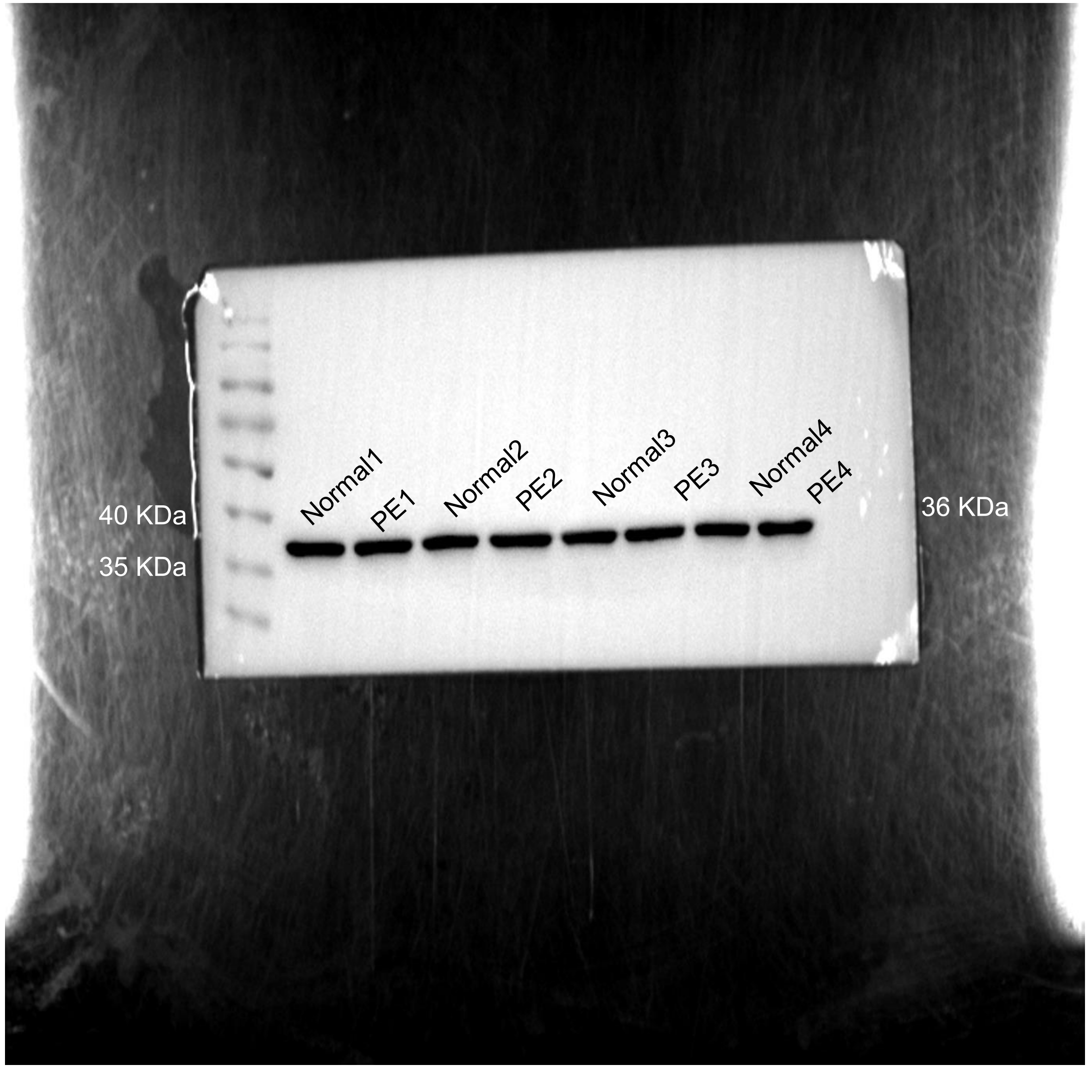

Figure1B GAPDH-3

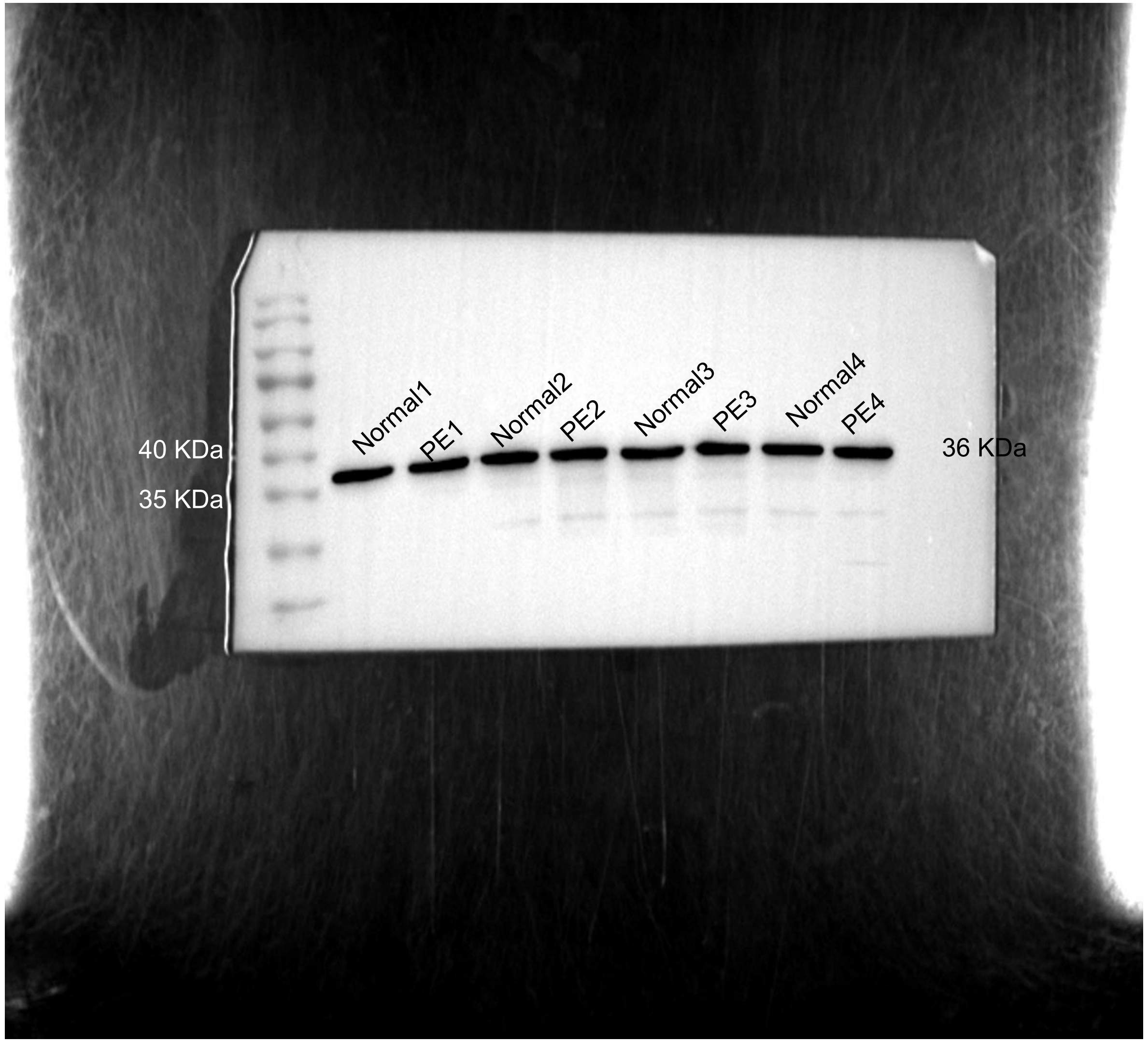

Figure2A: WTAP(as shown in this study)

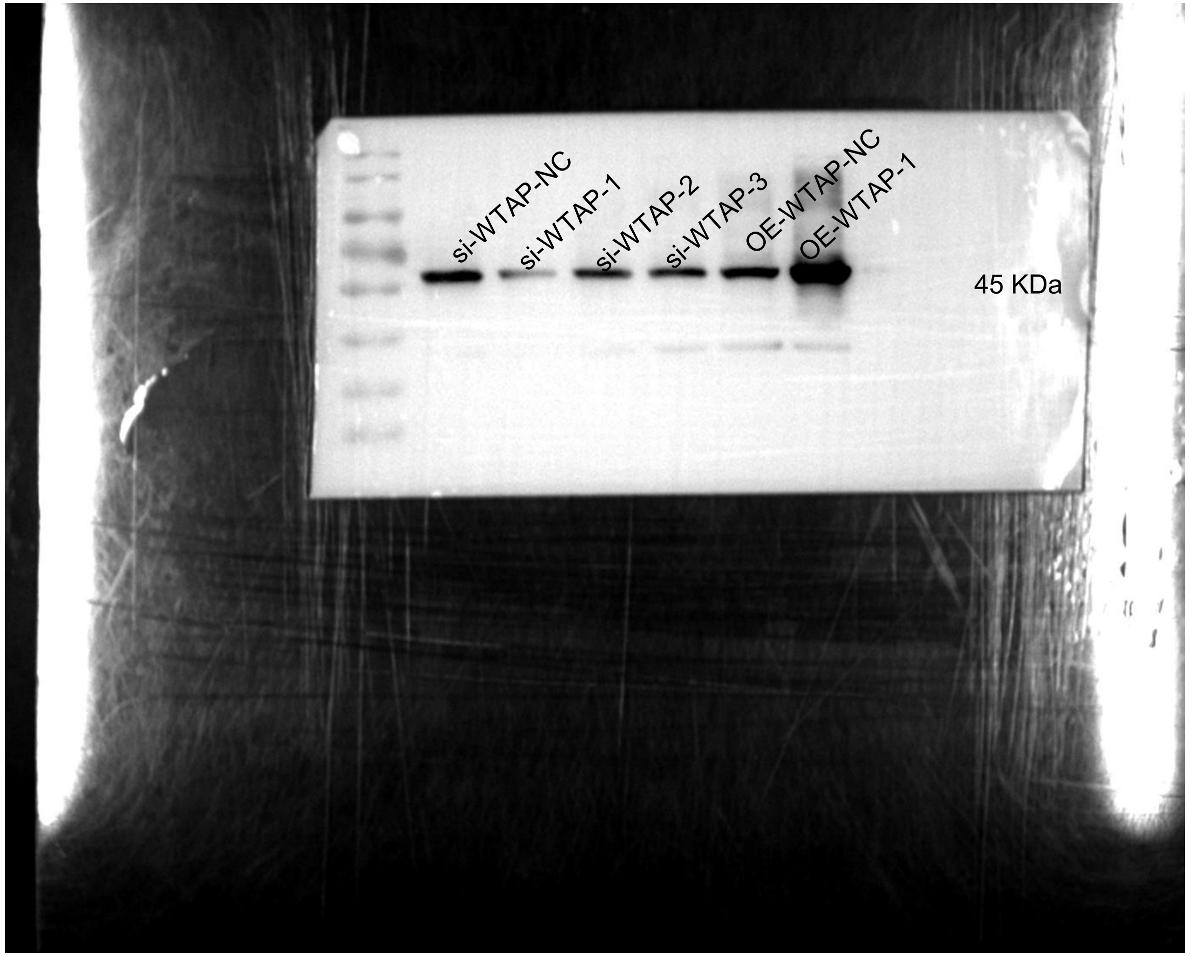

Figure2A: GAPDH(as shown in this study)

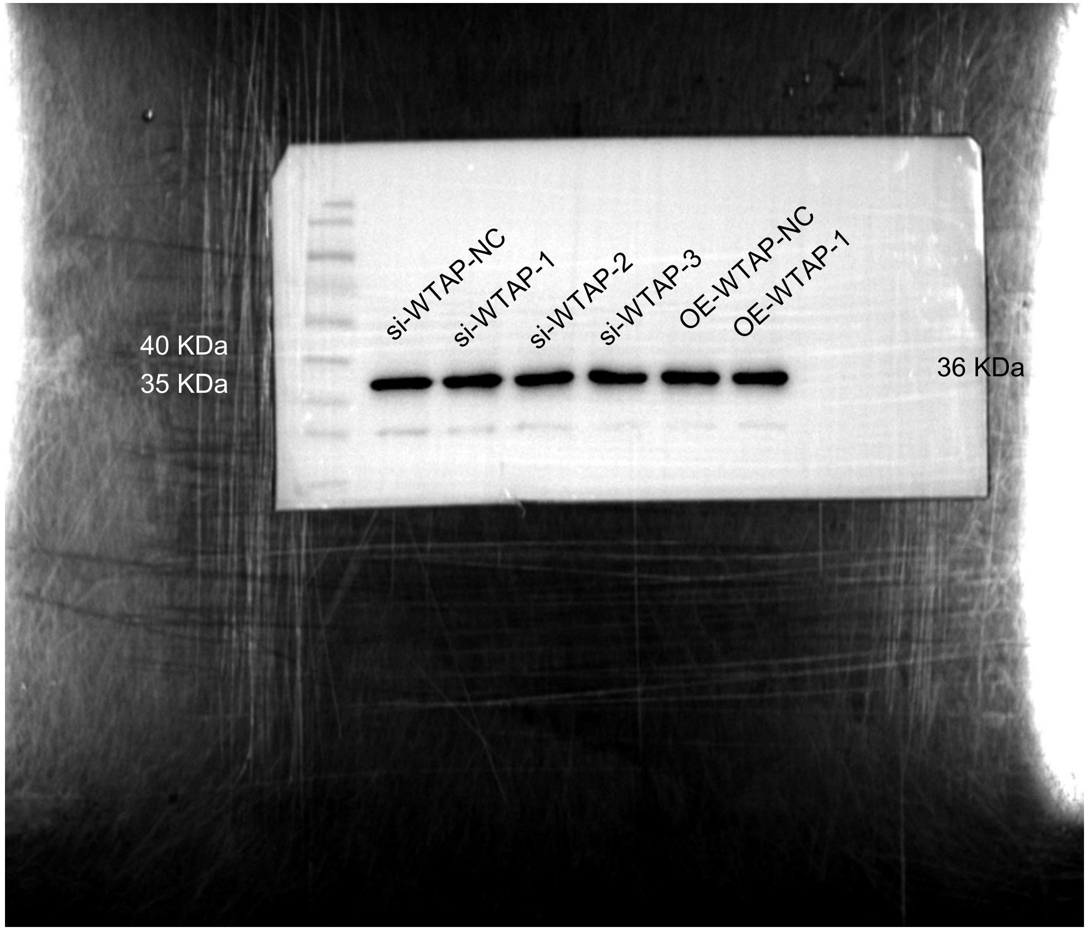

Figure2I :GPX4(as shown in this study)

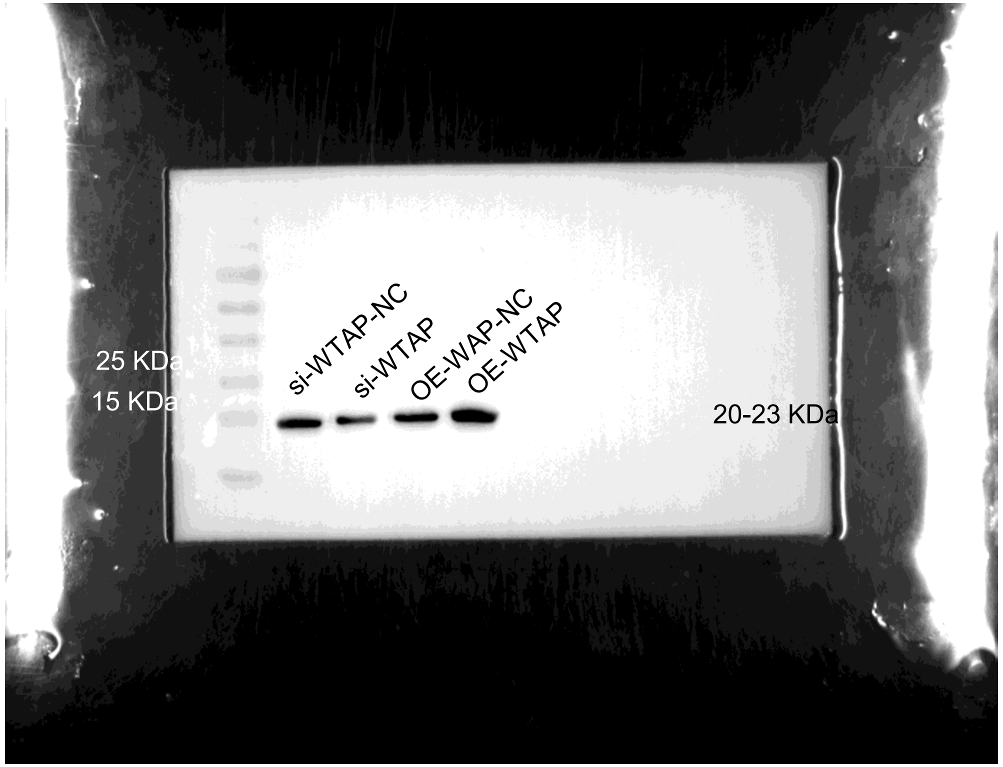

Figure2I: NRF2(as shown in this study)

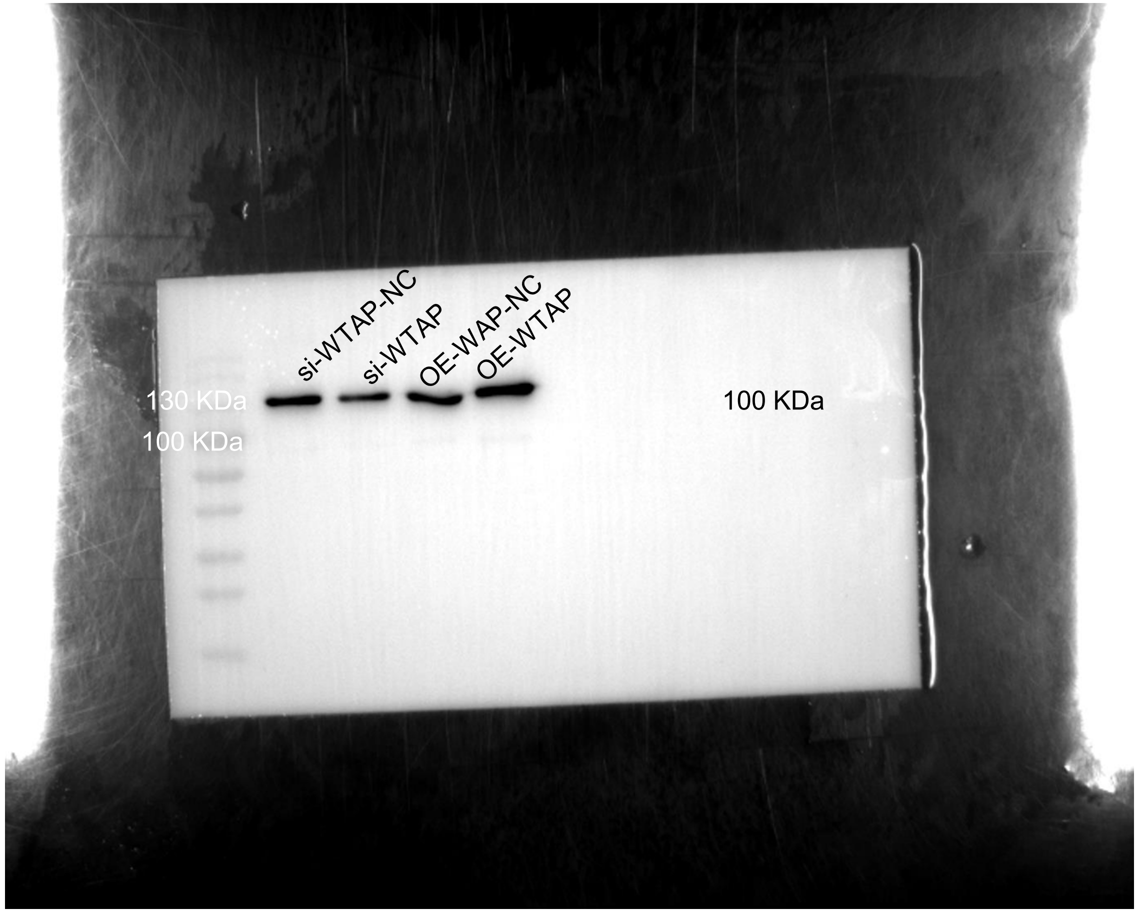

Figure2I: GAPDH(as shown in this study)

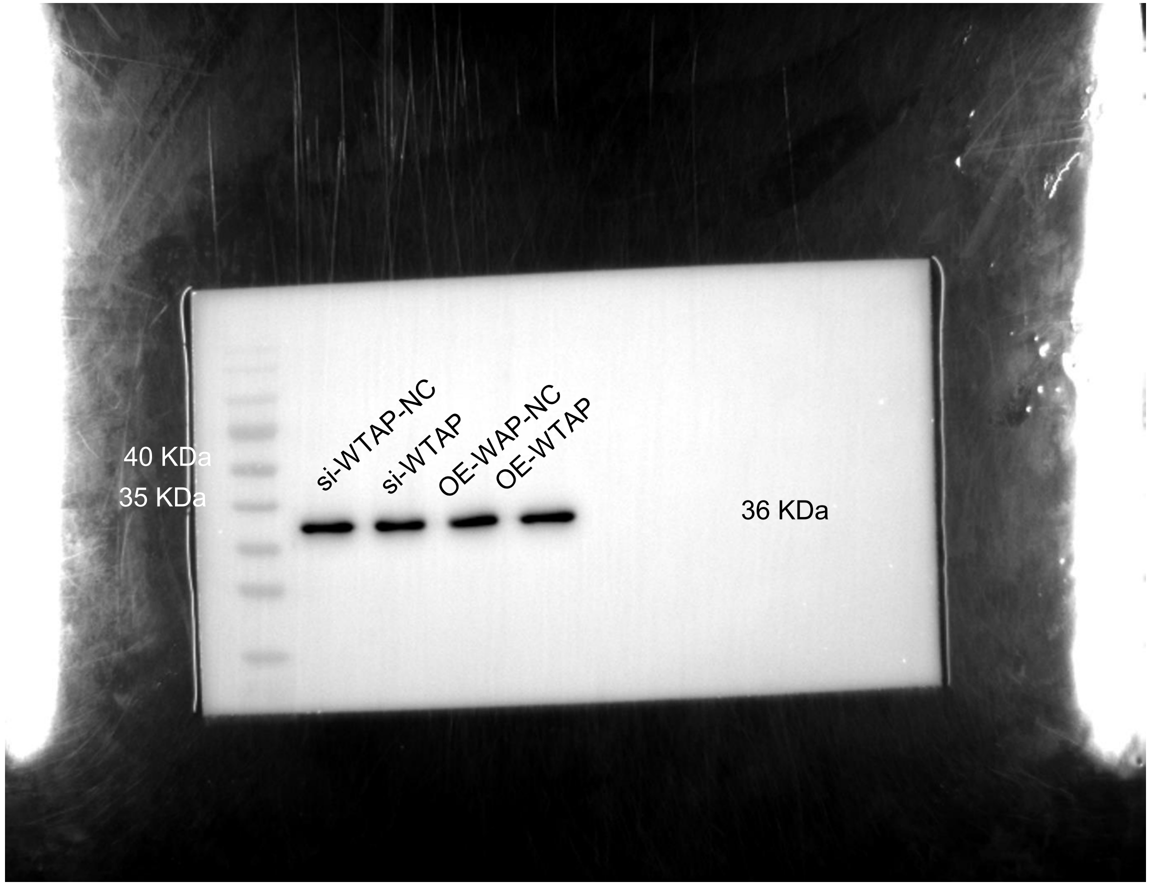

Figure3D YTHDF2-1(as shown in this study)

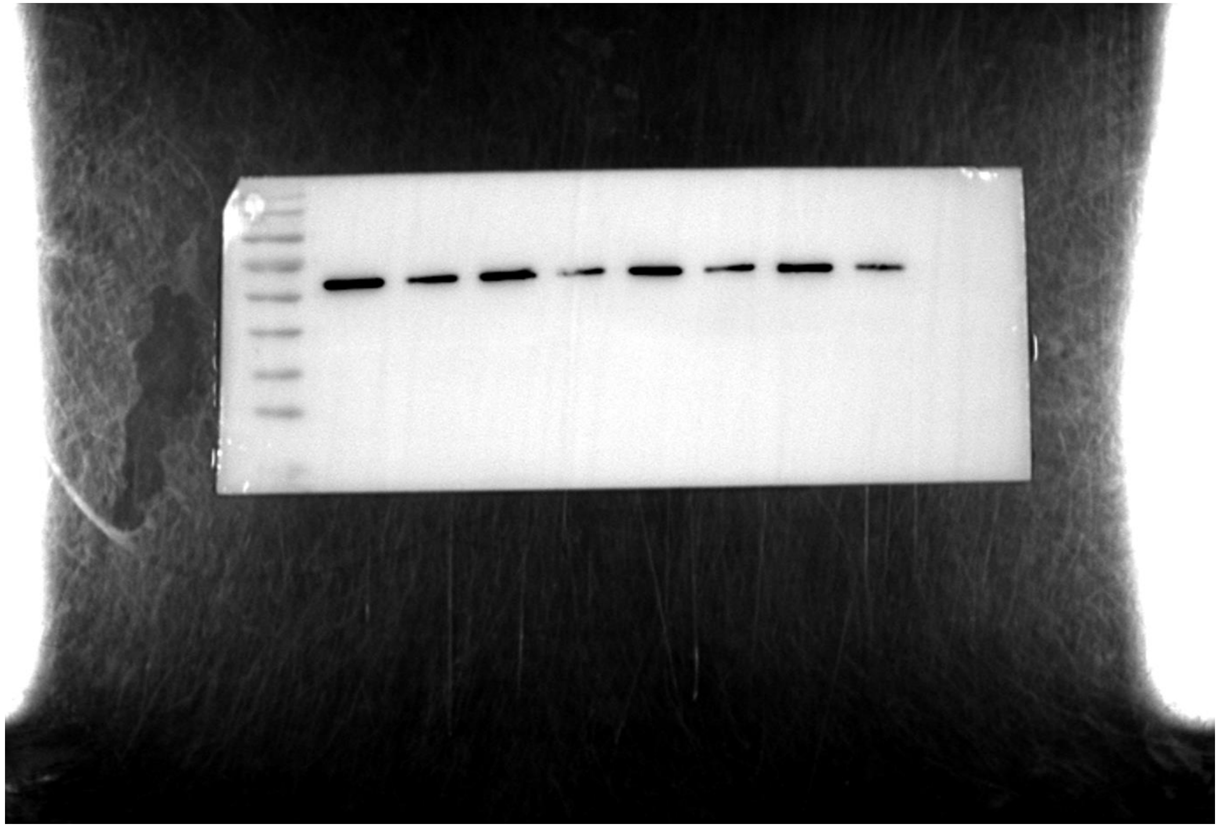

Figure3D YTHDF2-2

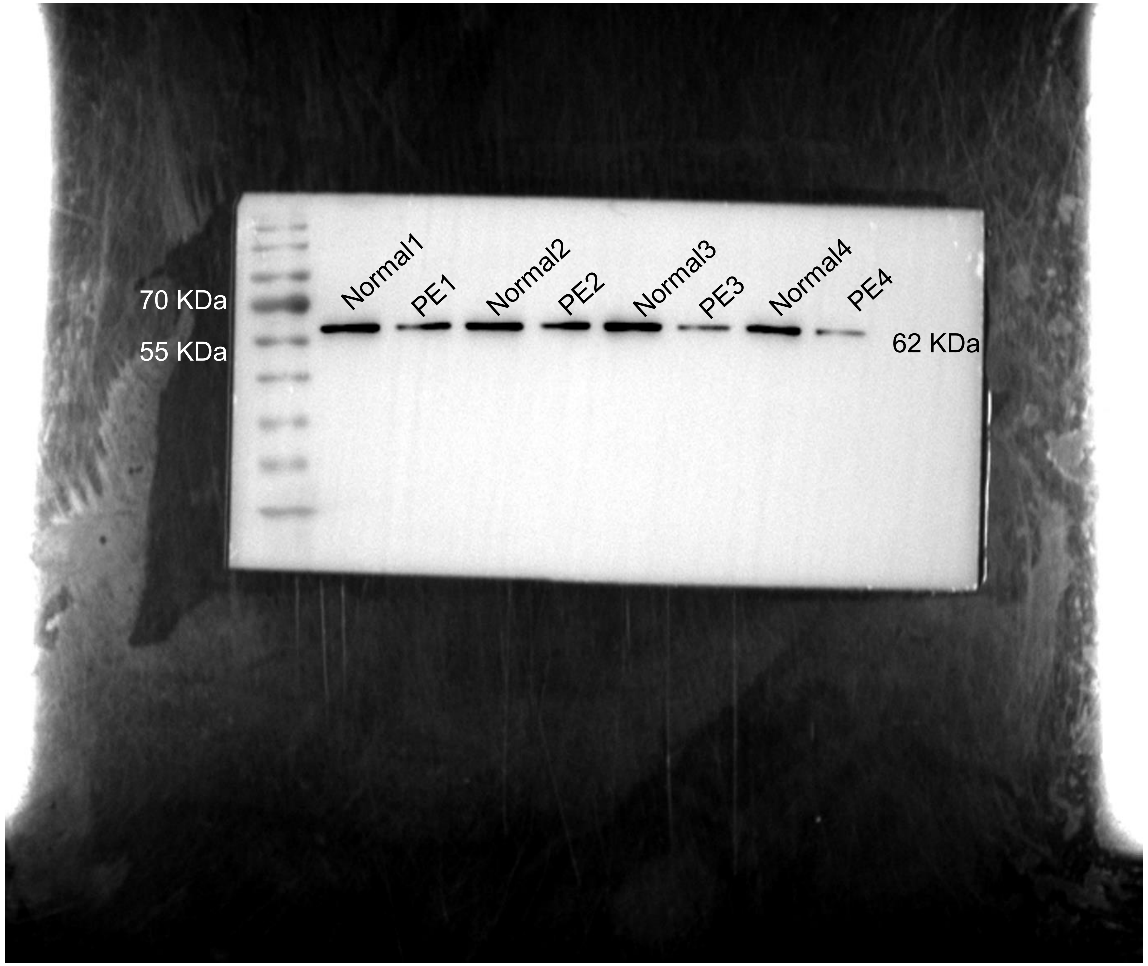

Figure3D YTHDF2-3

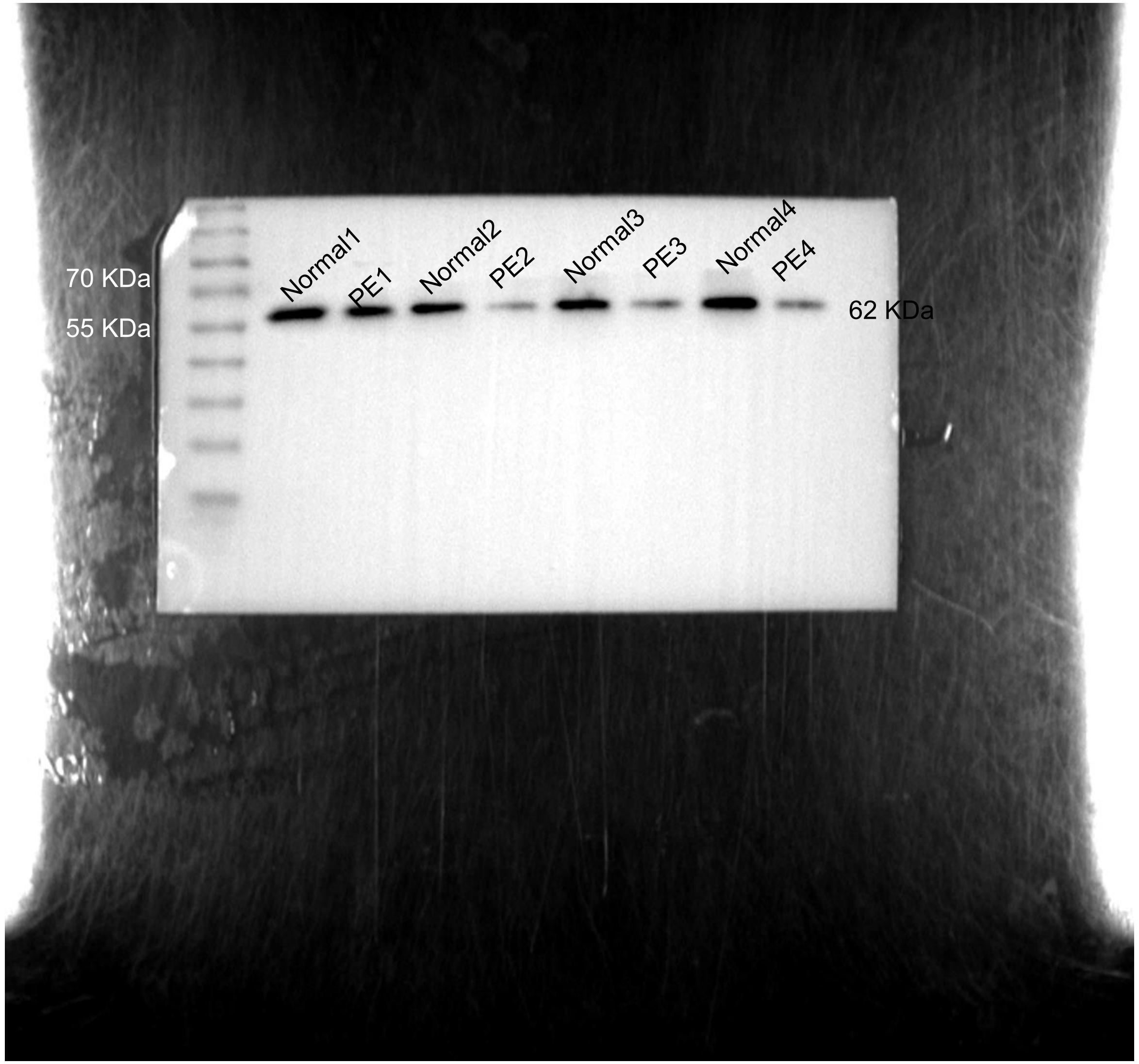

Figure3D YTHDF2-4

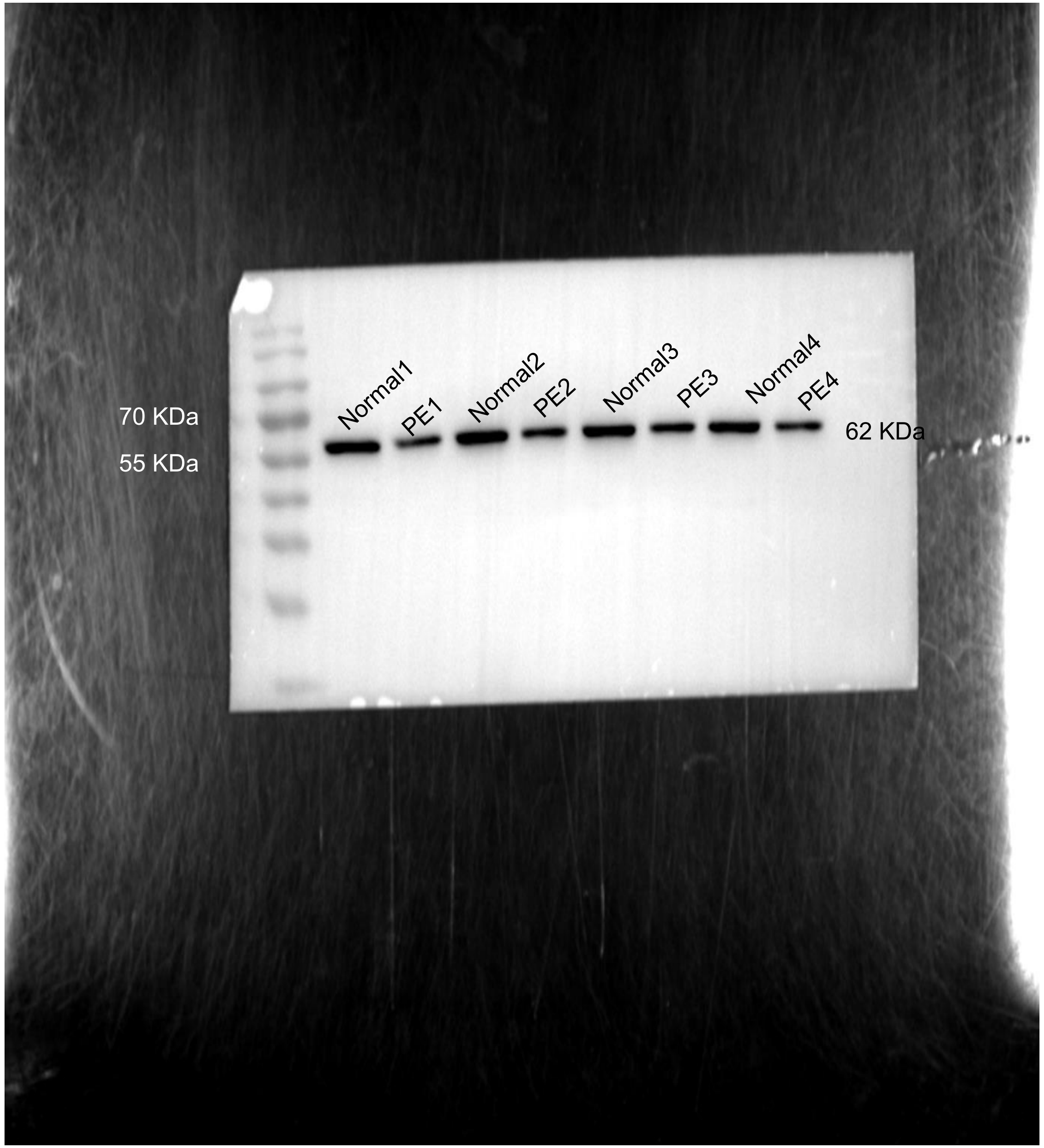

Figure3D GAPDH-1(as shown in this study)

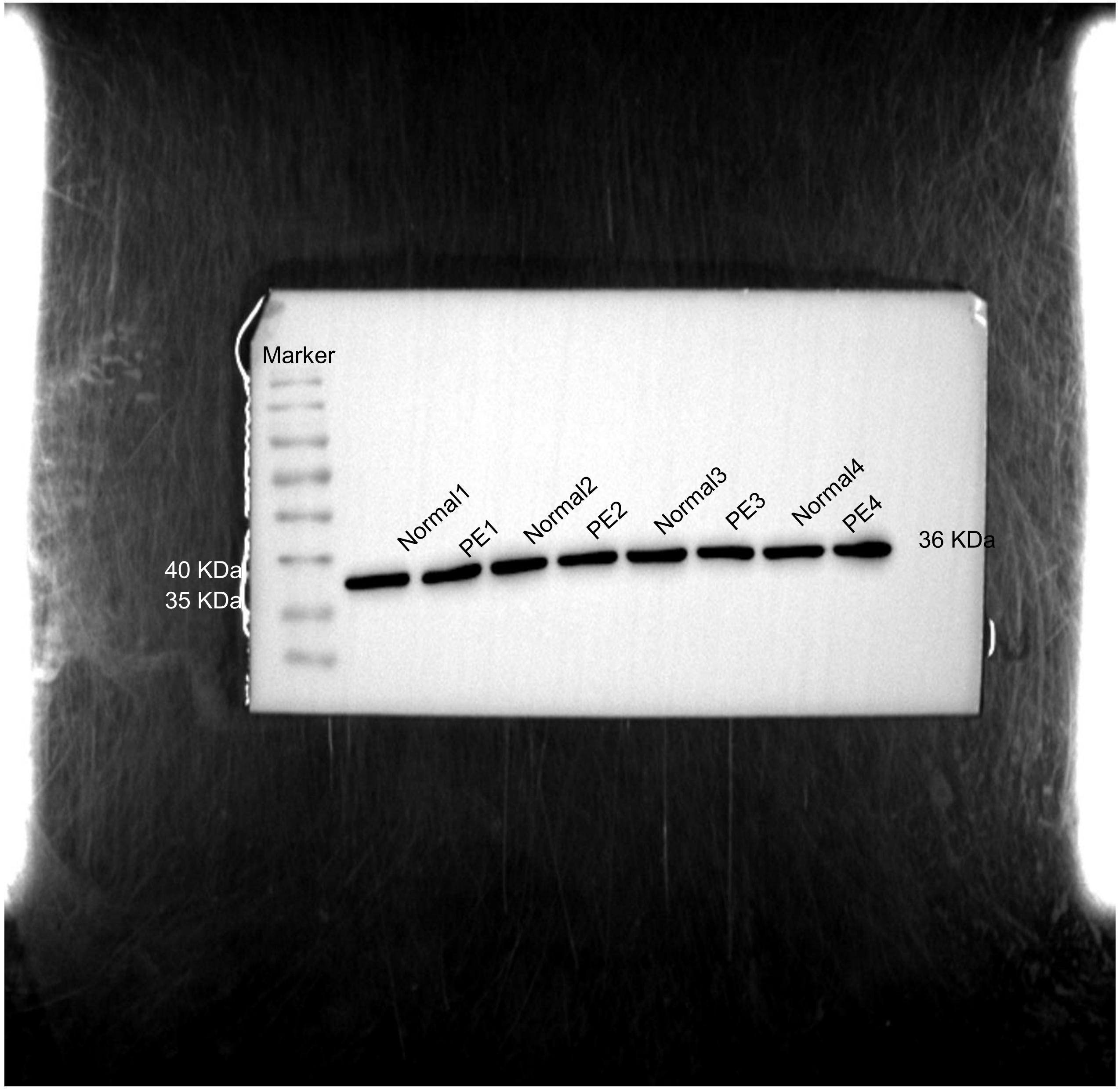

Figure3D GAPDH-2

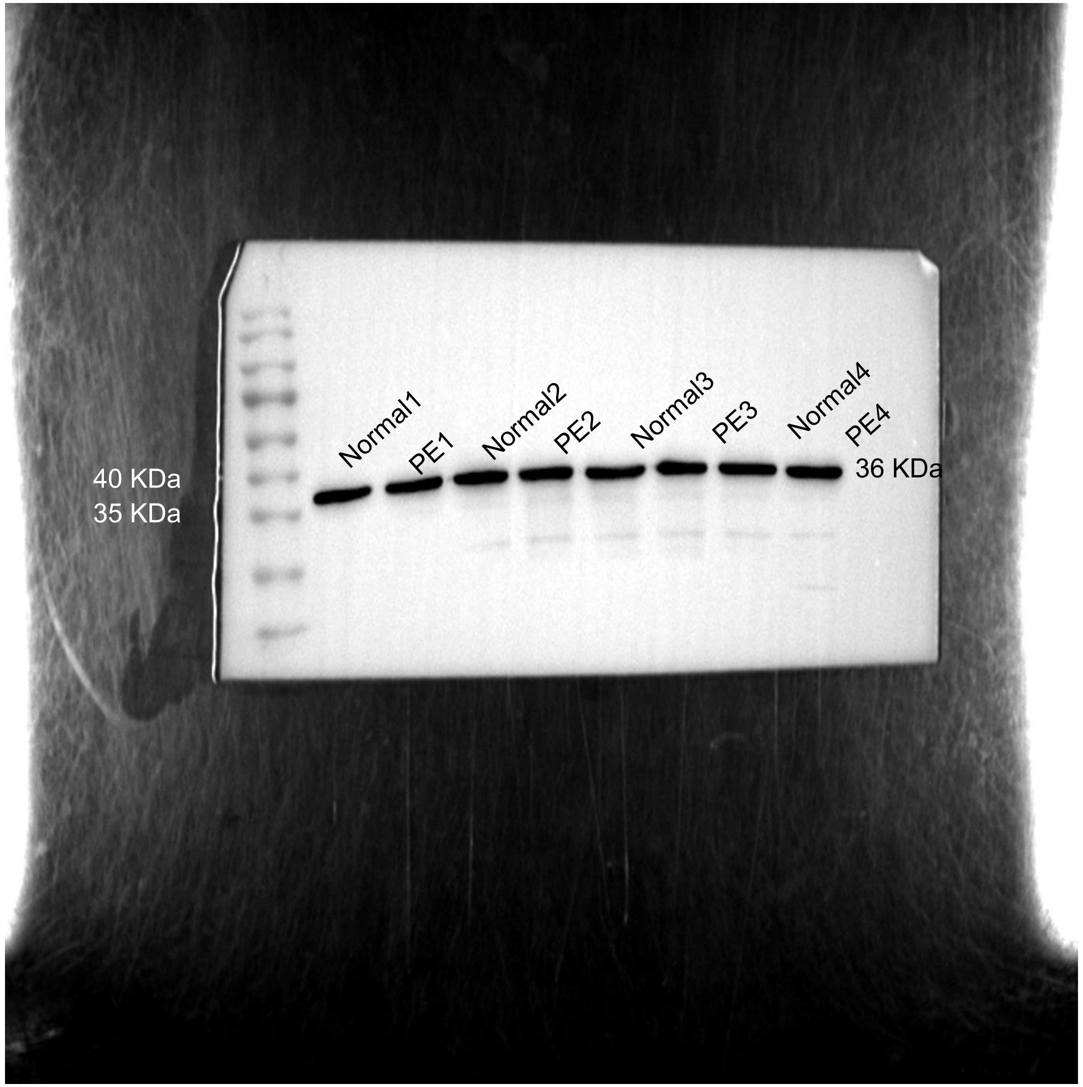

Figure3D GAPDH-3

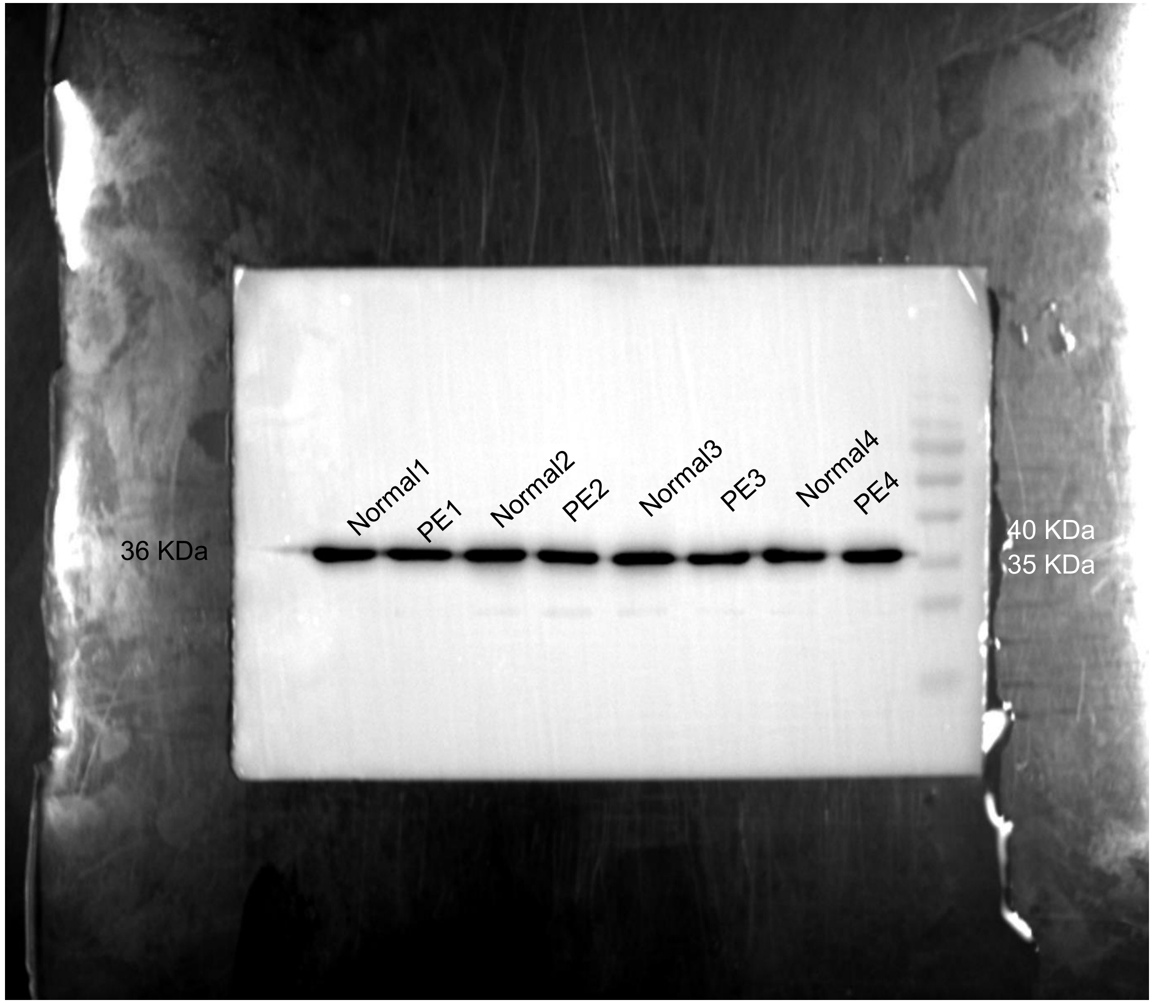

Figure3F NCOA4-1

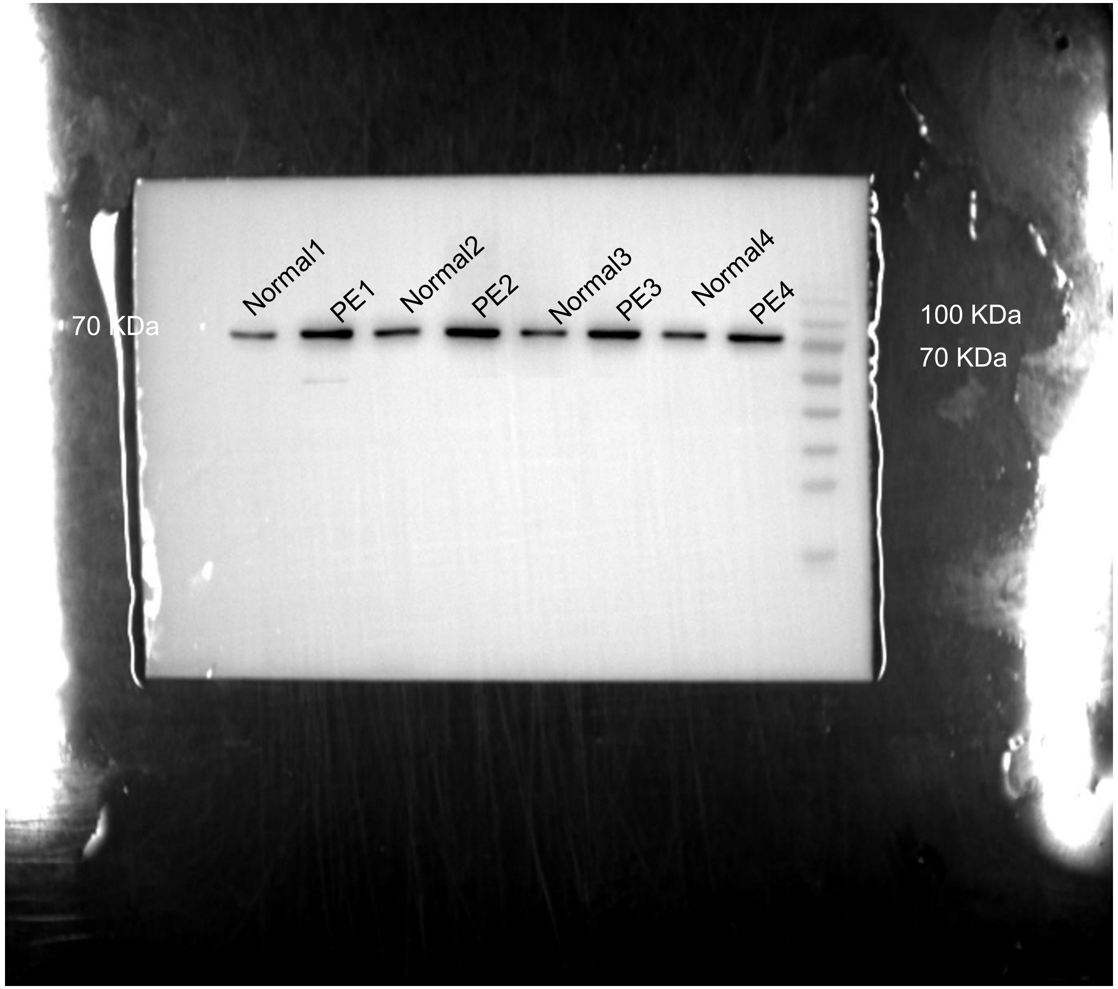

Figure3F NCOA4-2(as shown in this study)

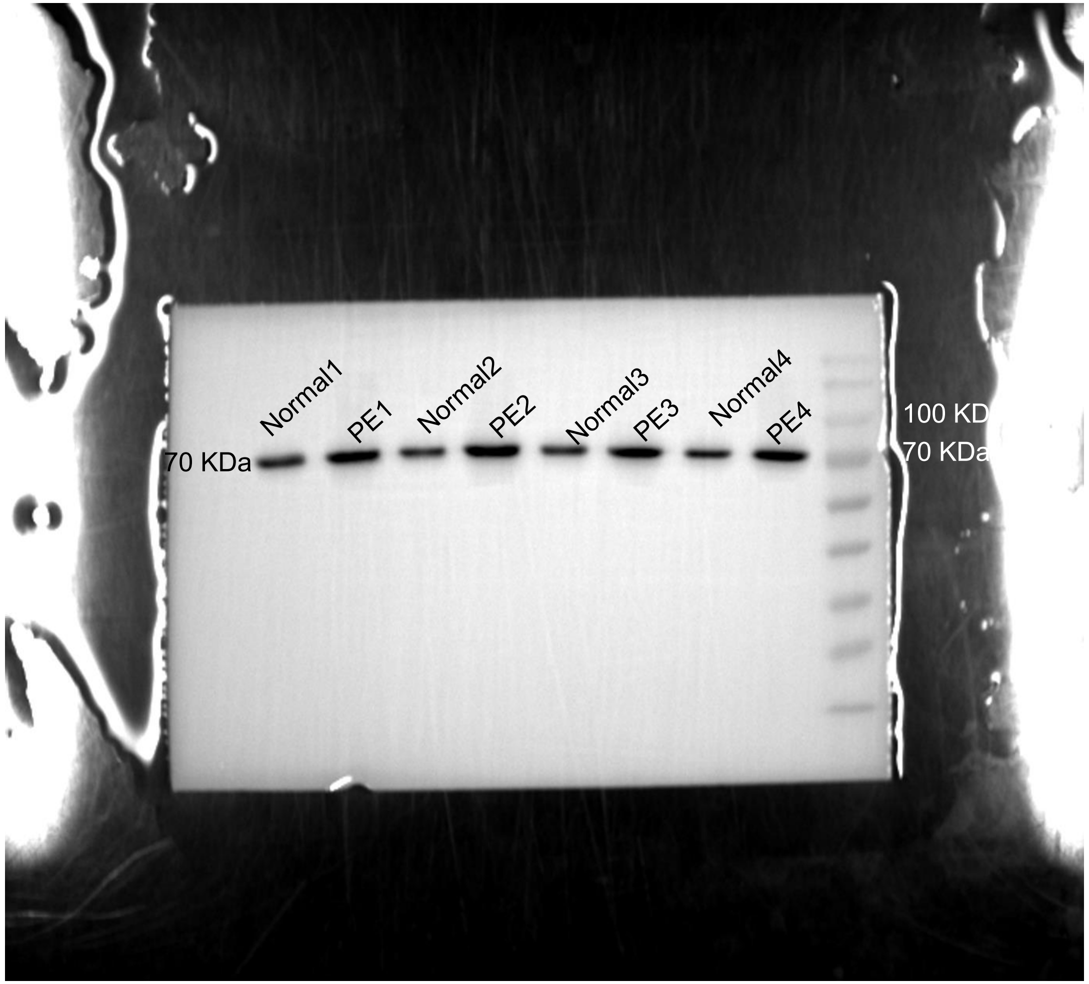

Figure3F NCOA4-3

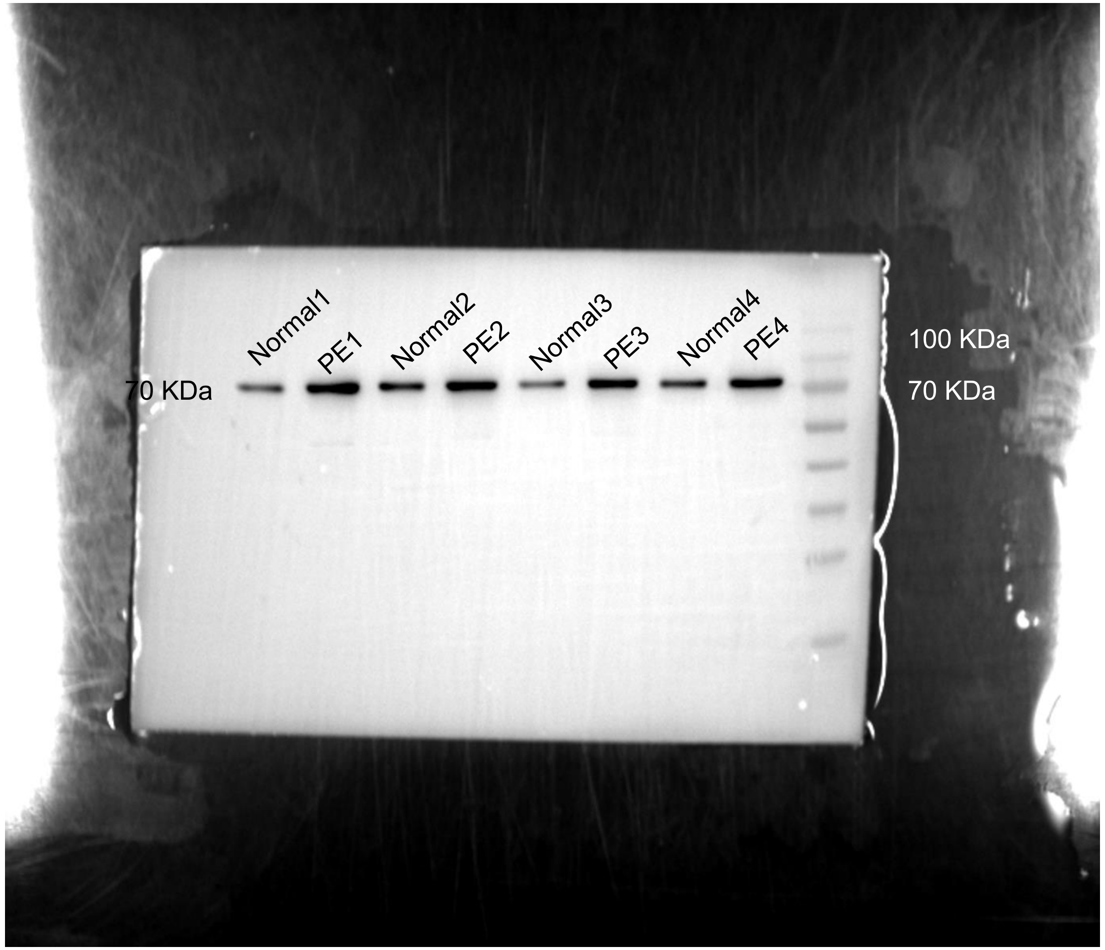

Figure3F GAPDH-1(as shown in this study)

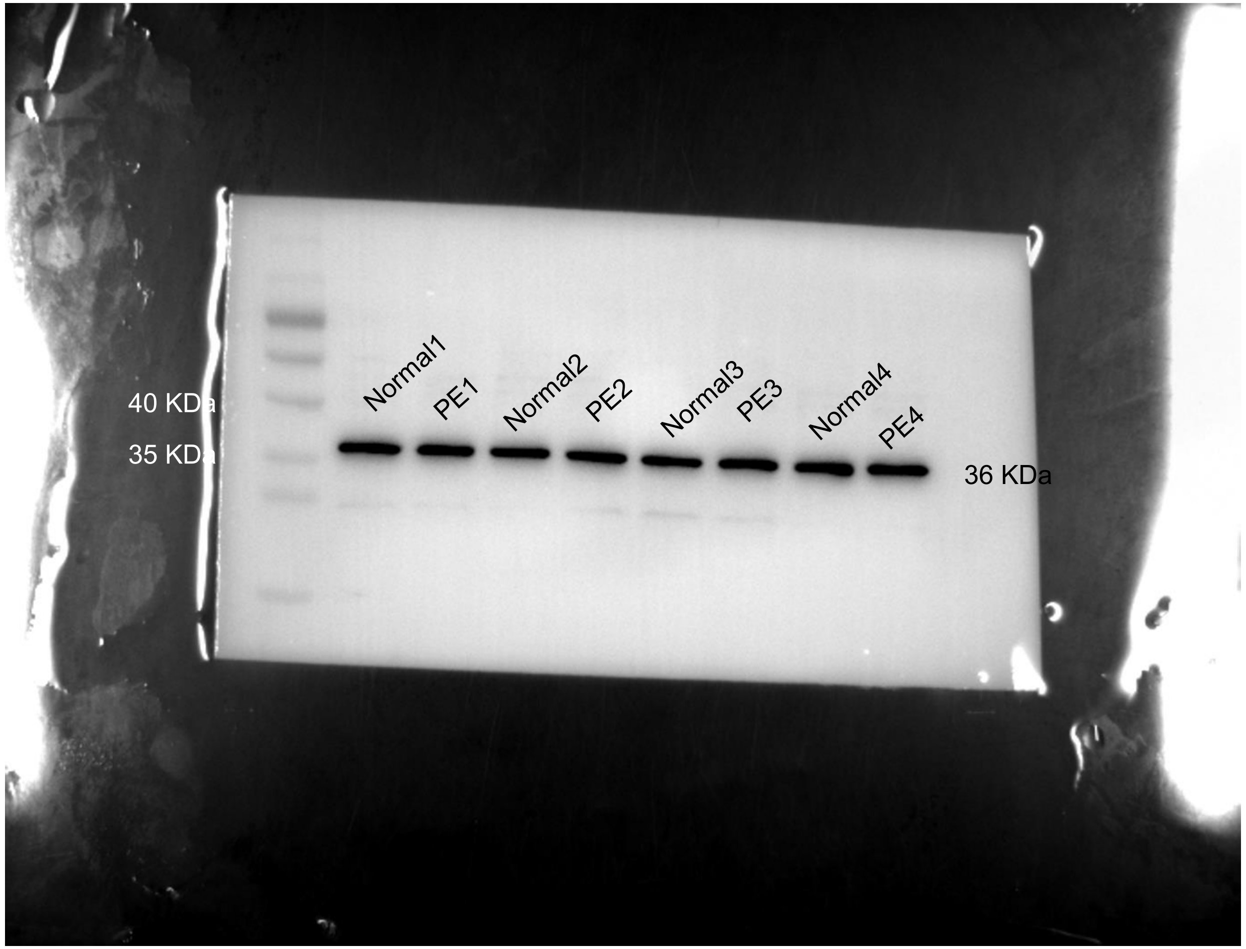

Figure3F GAPDH-2

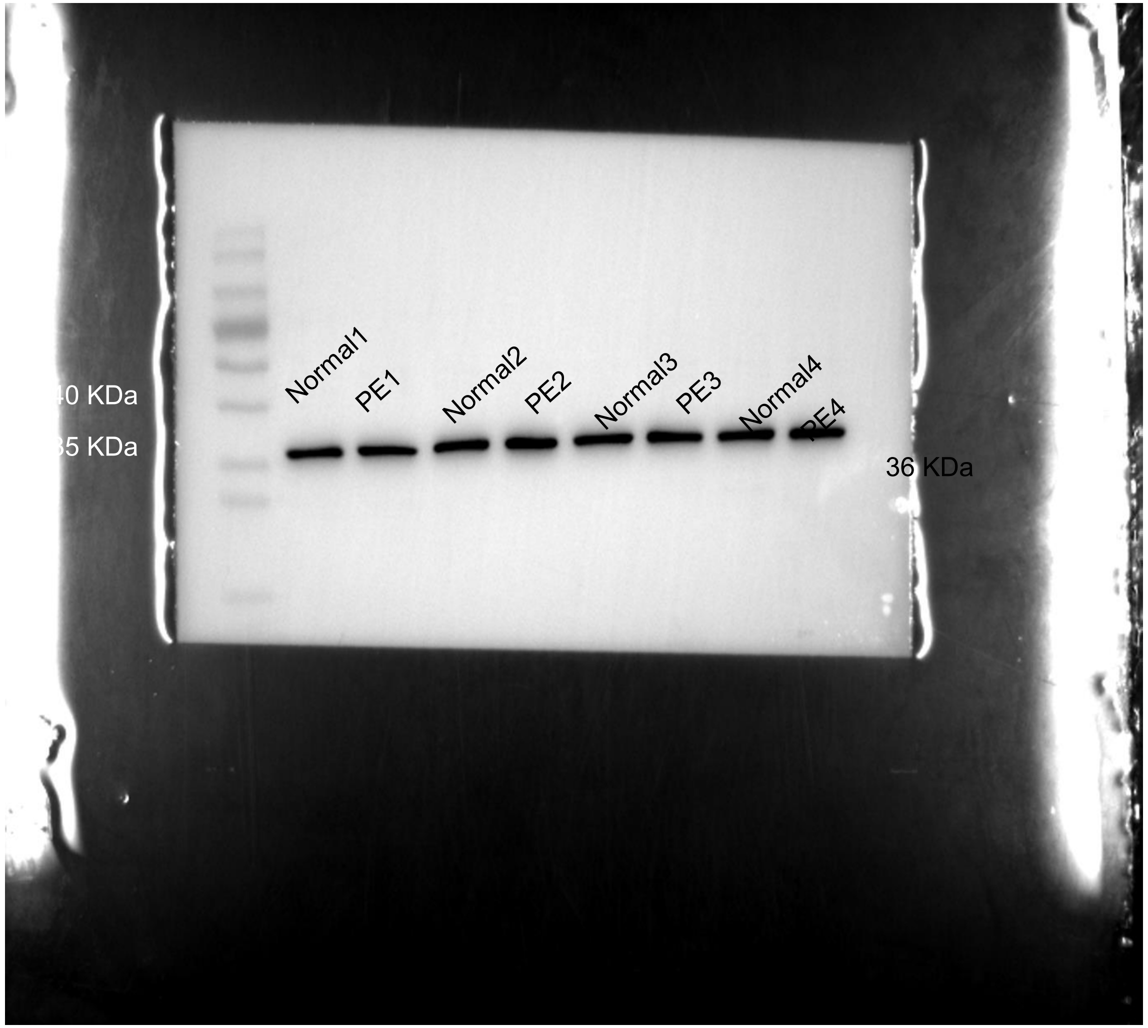

Figure3F GAPDH-3

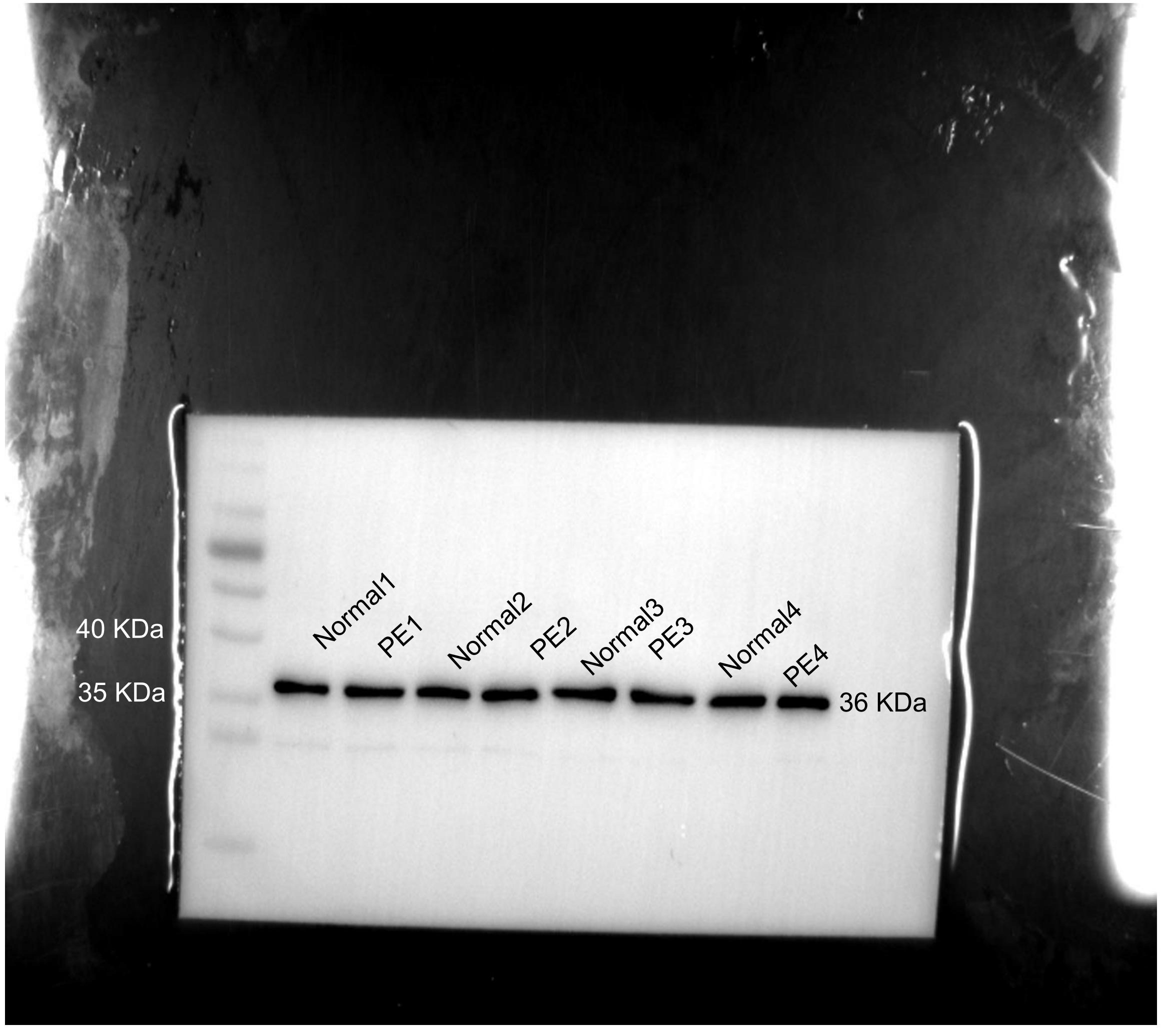

Figure4B NCOA4-1(as shown in this study)

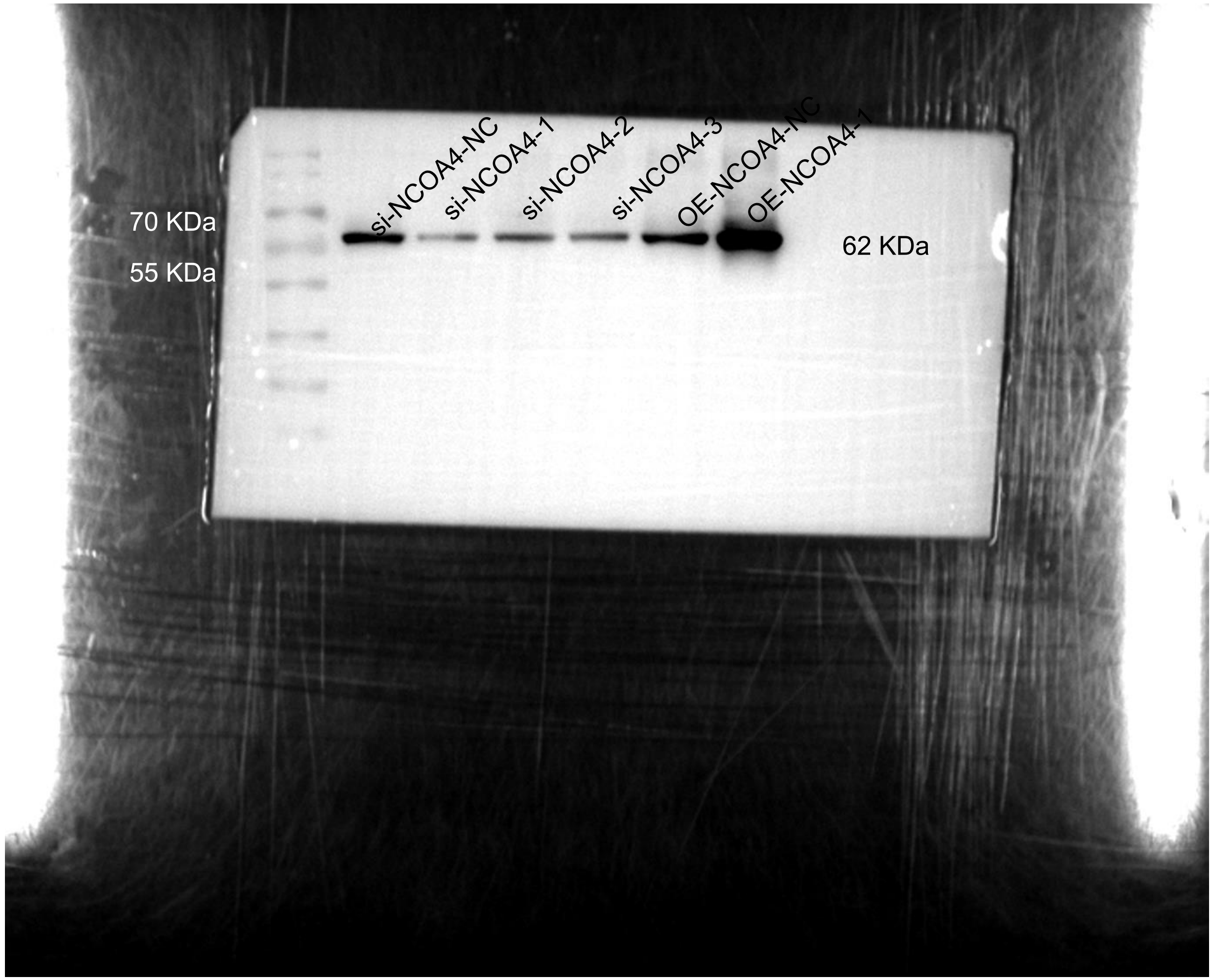

Figure4B GAPDH-1(as shown in this study)

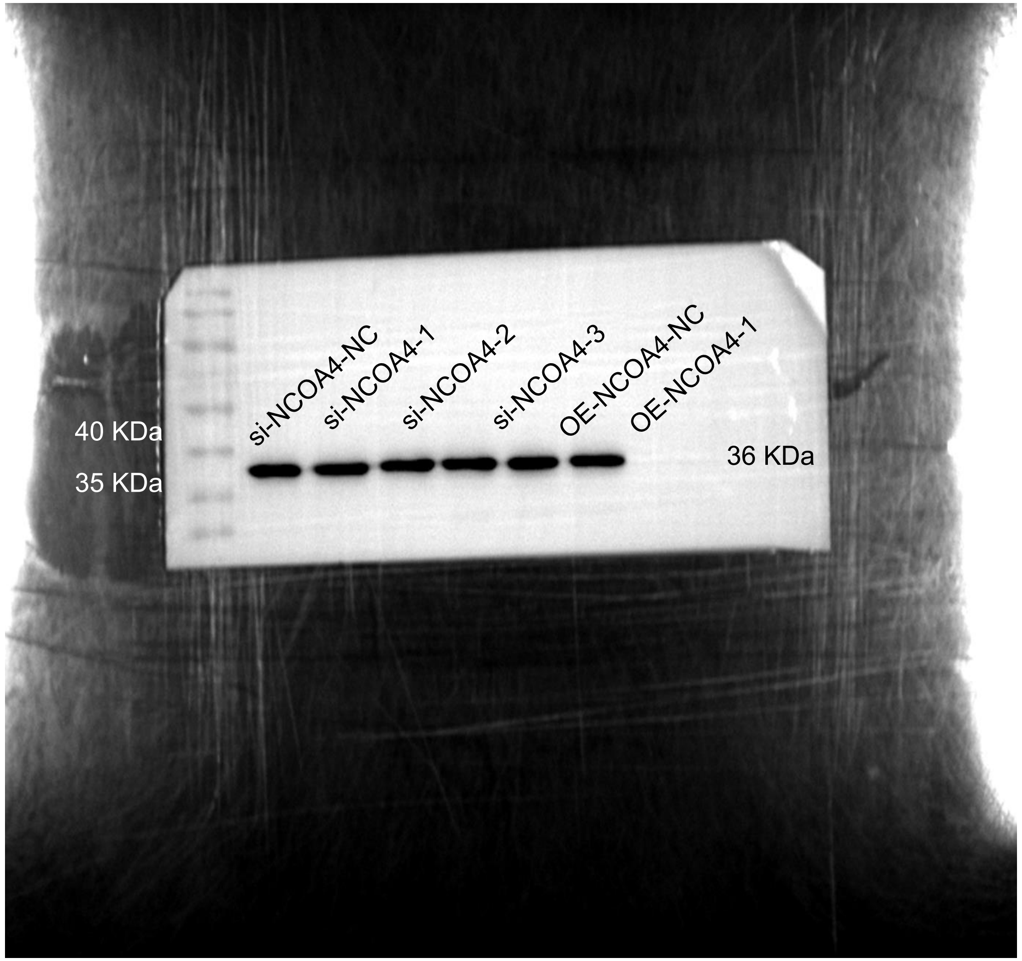

Figure4J NCOA4-1(as shown in this study)

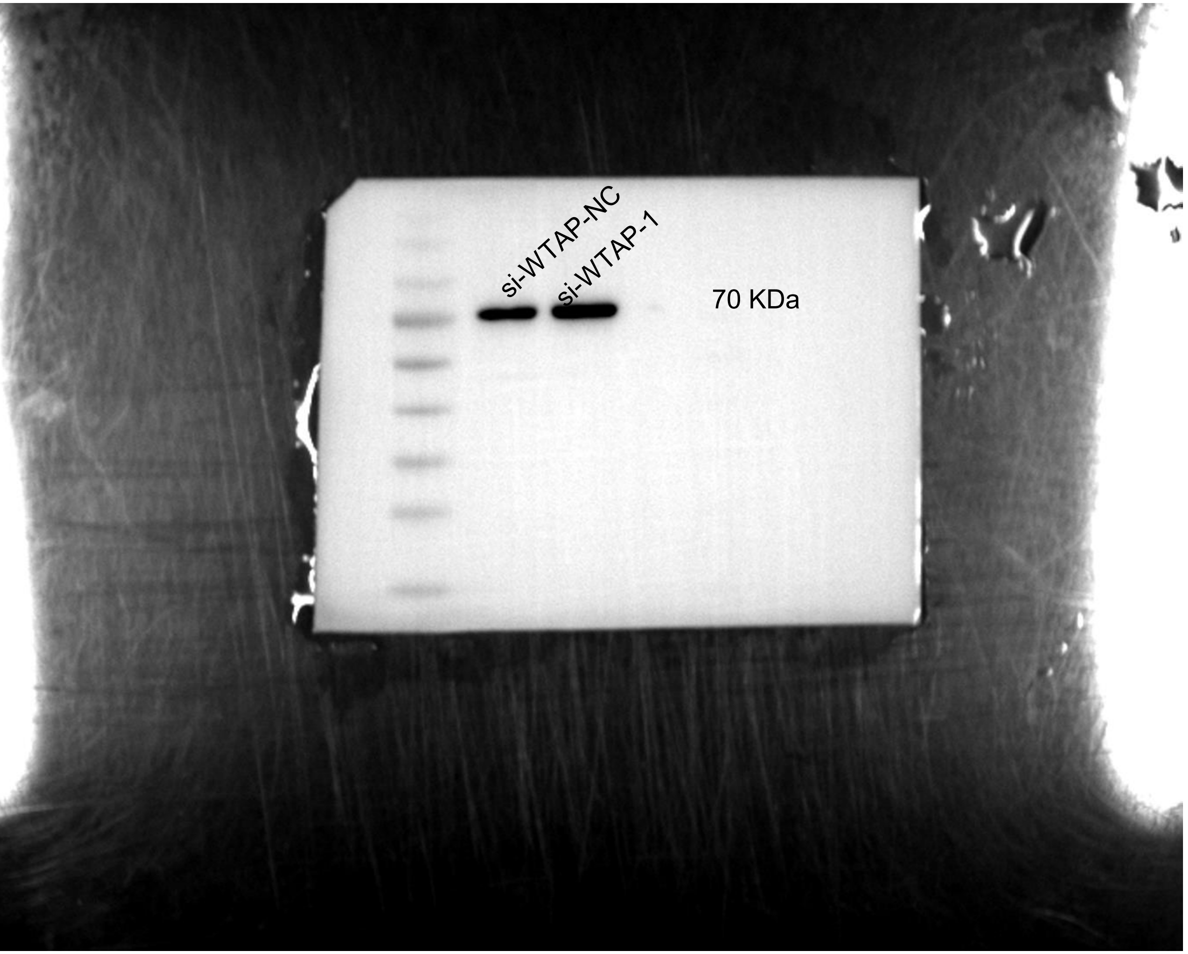

Figure4J NCOA4-2

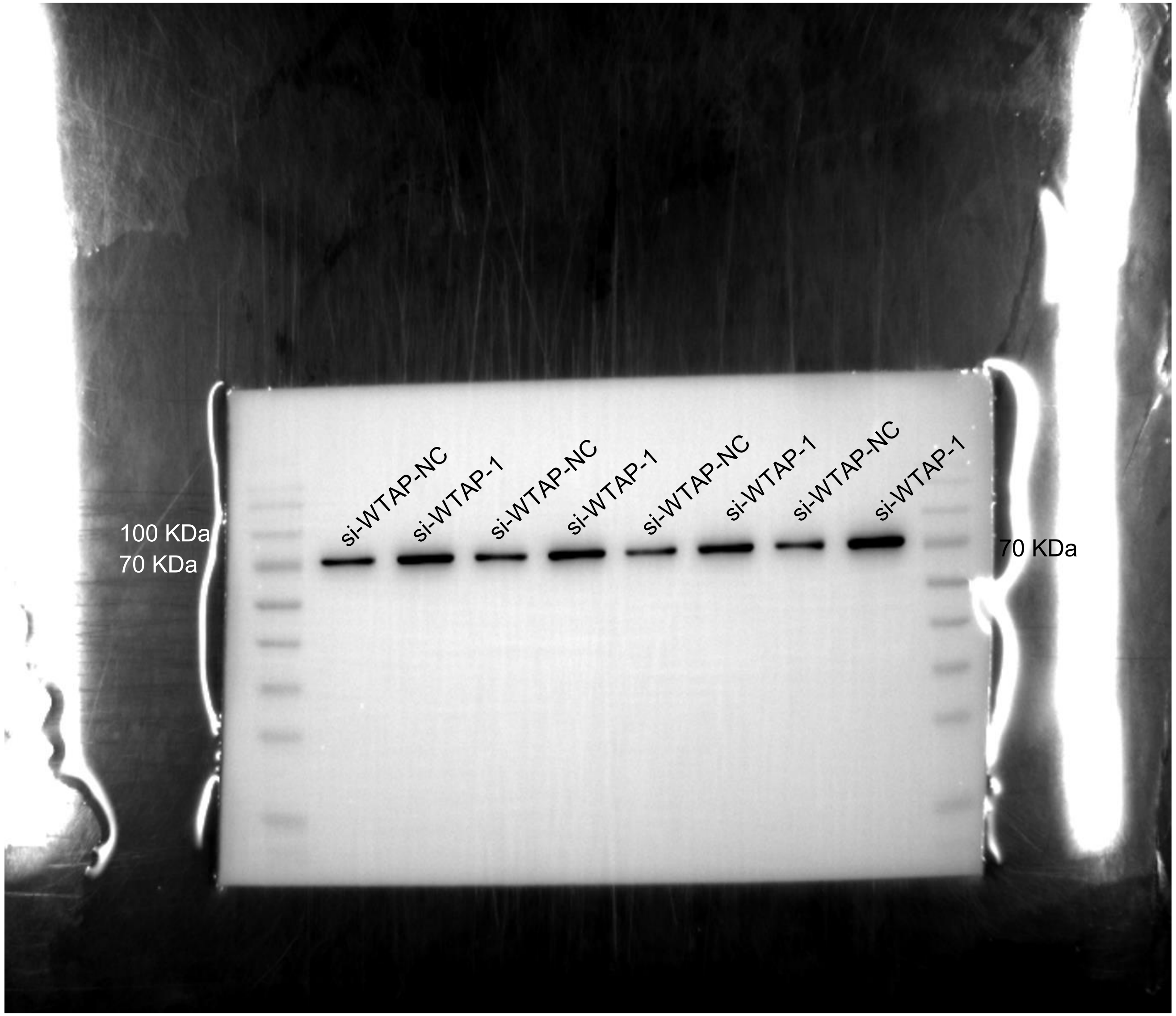

Figure4J GAPDH-1(as shown in this study)

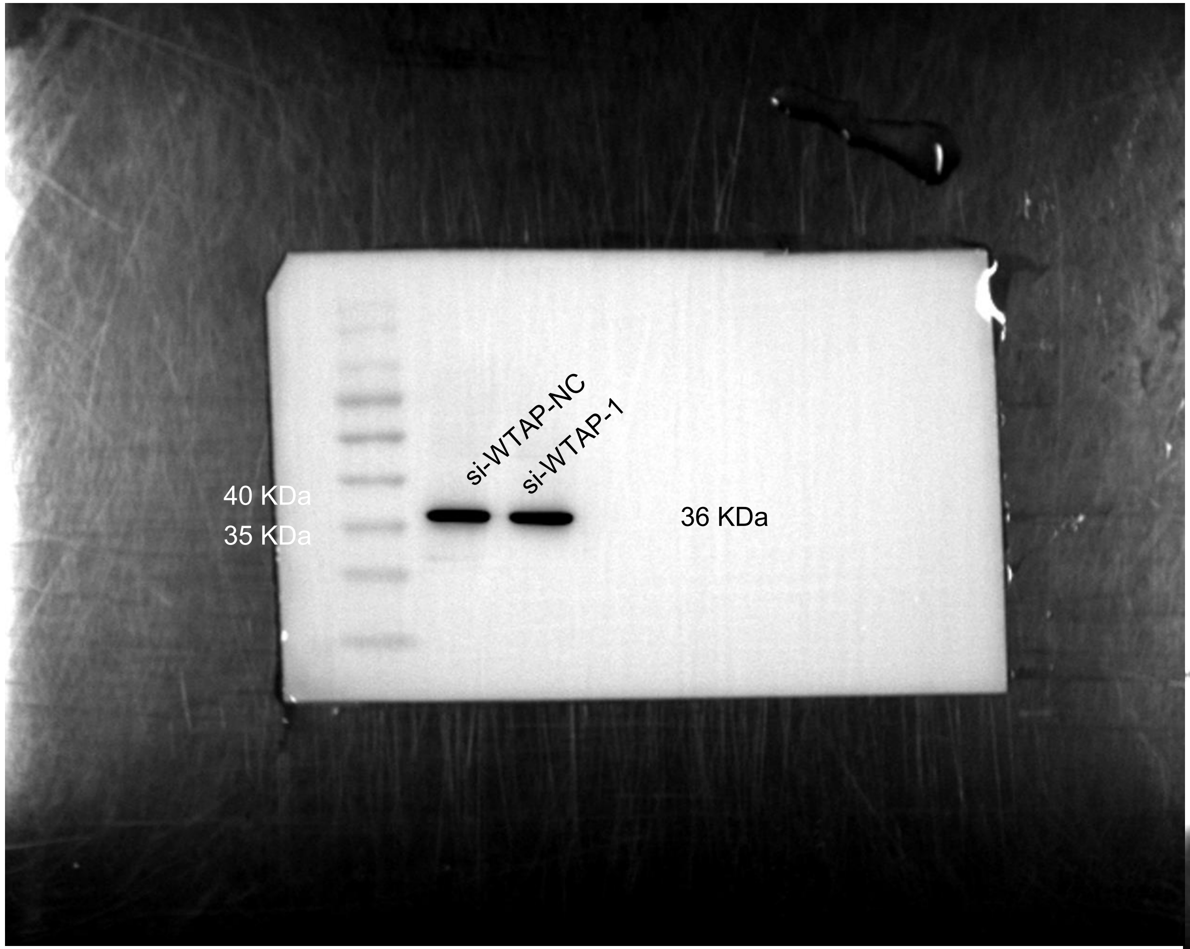

Figure4J GAPDH-2

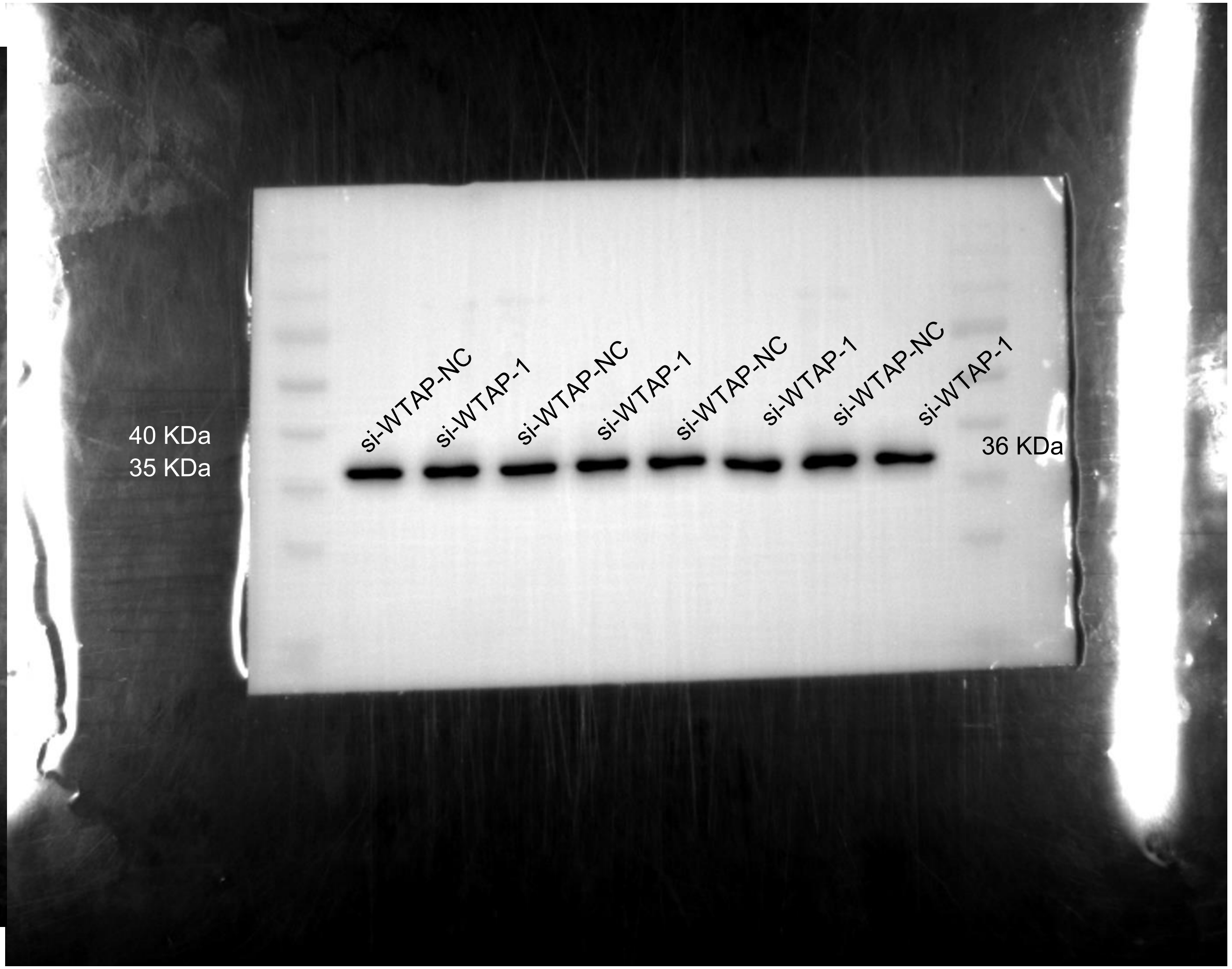

Figure5B YTHDF2(as shown in this study)

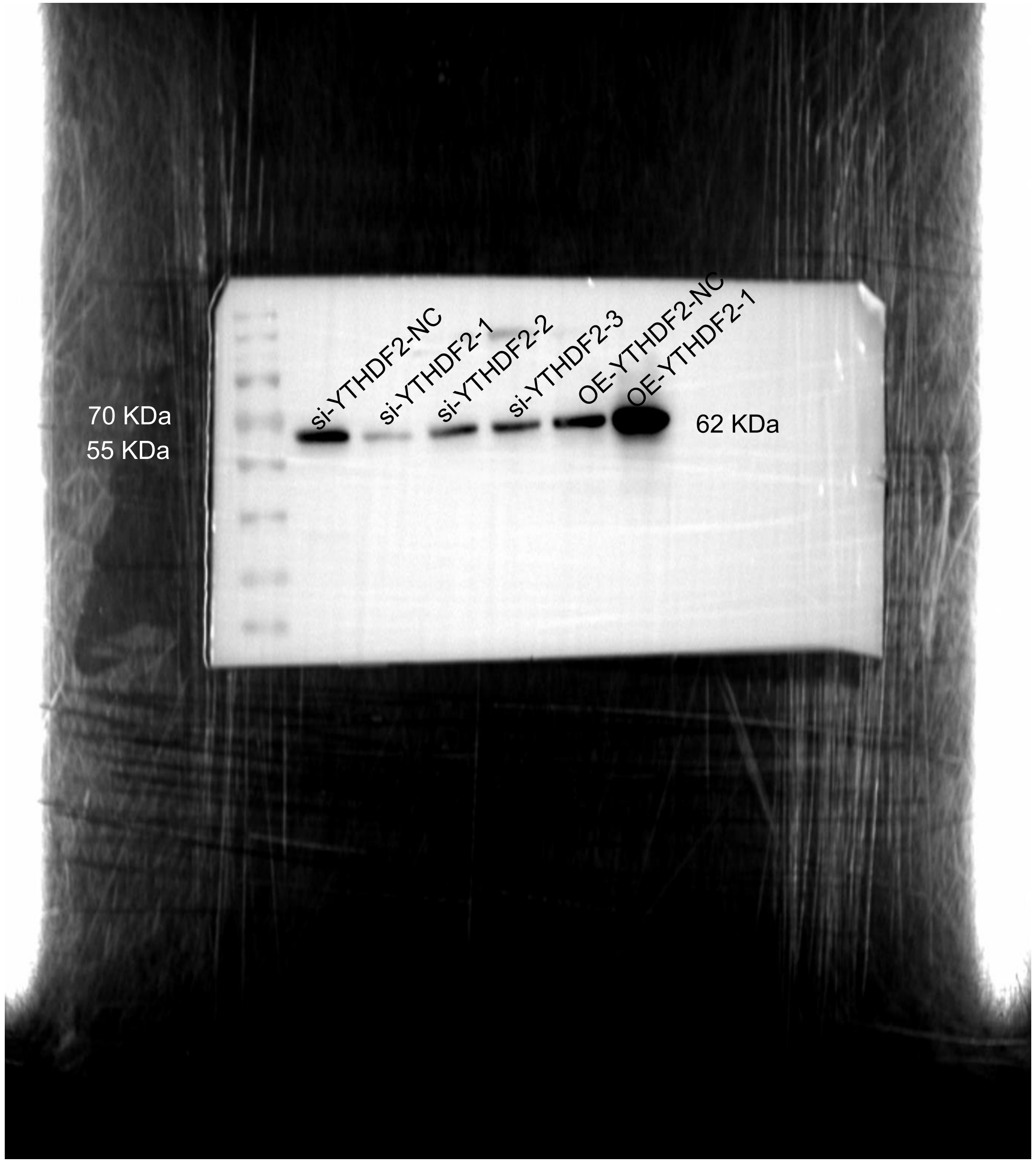

Figure5B GAPDH(as shown in this study)

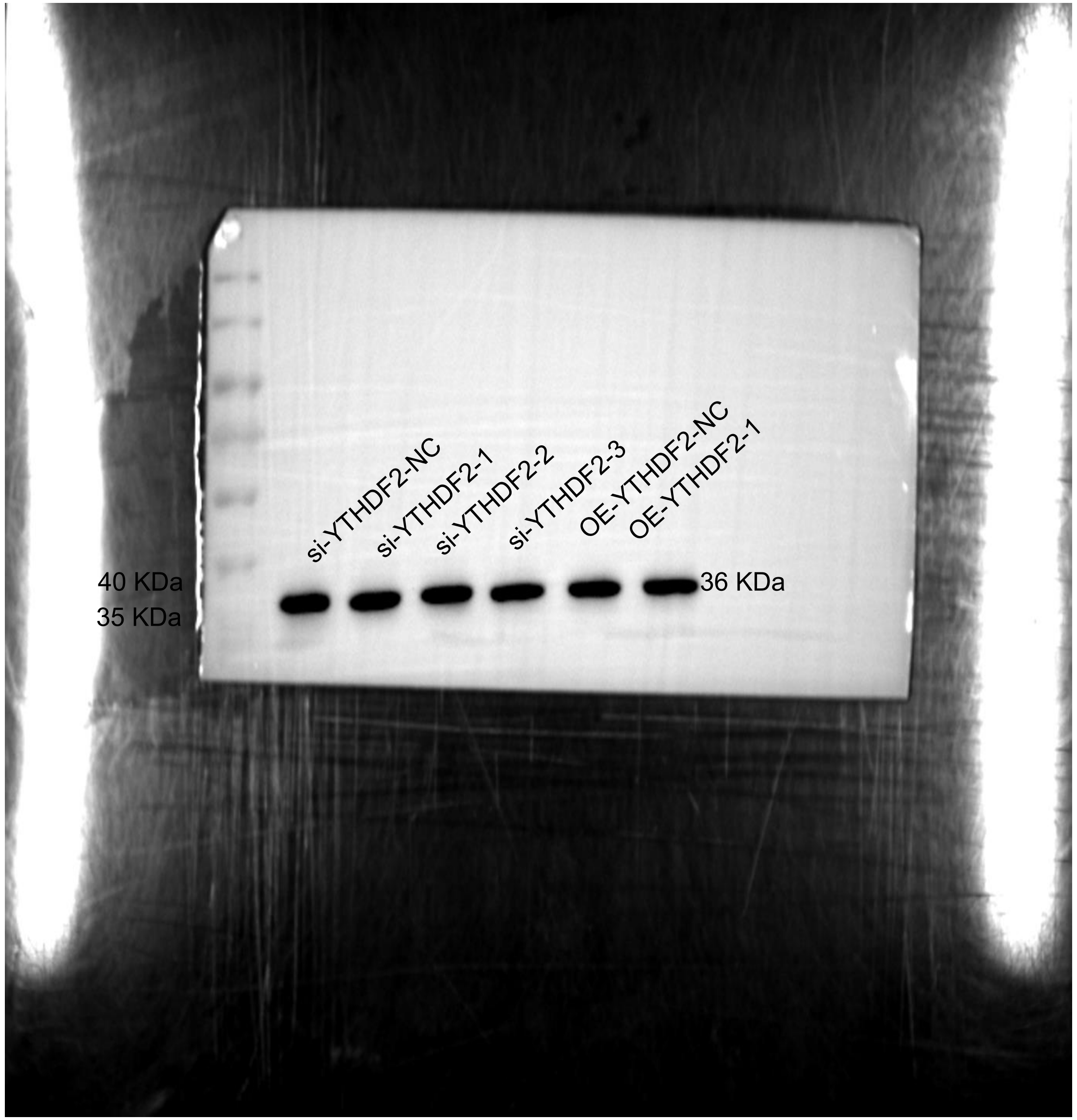

Figure5G NCOA4(as shown in this study)

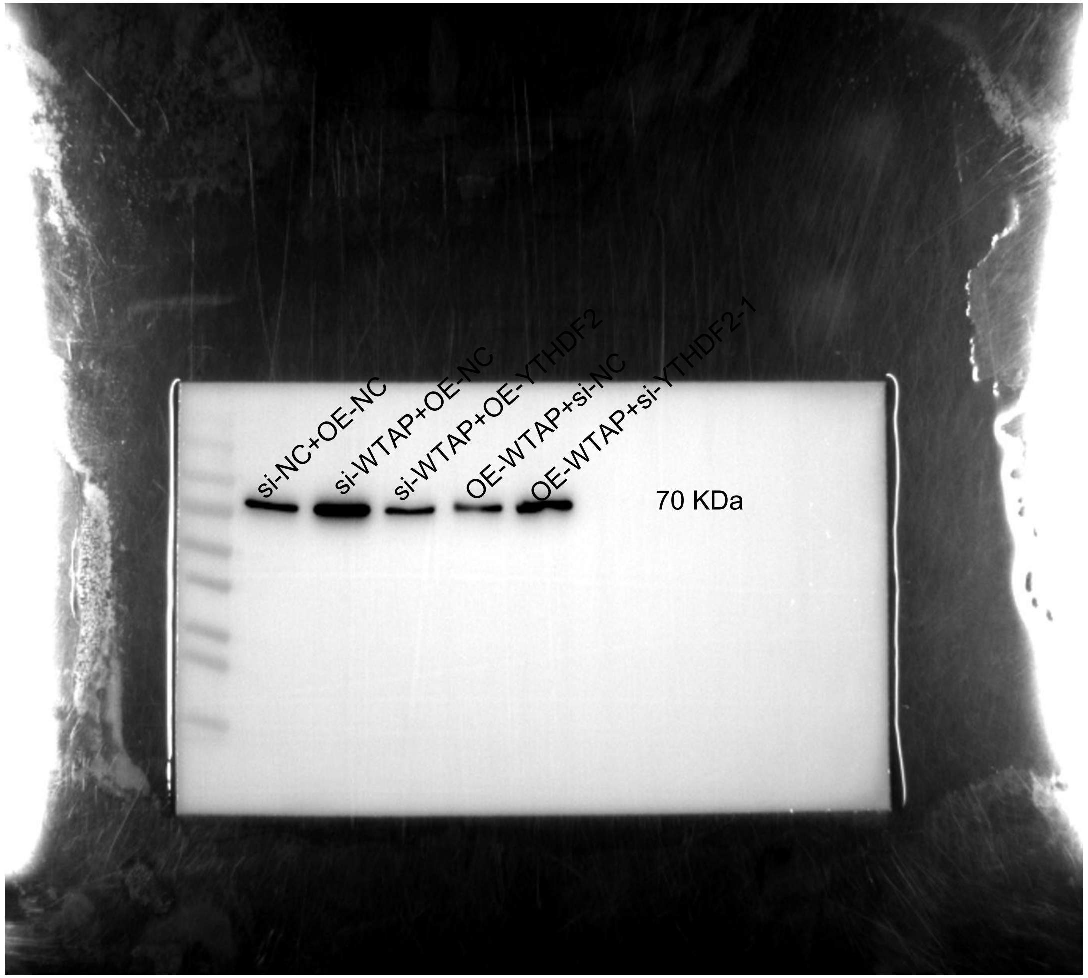

Figure5G GAPDH(as shown in this study)

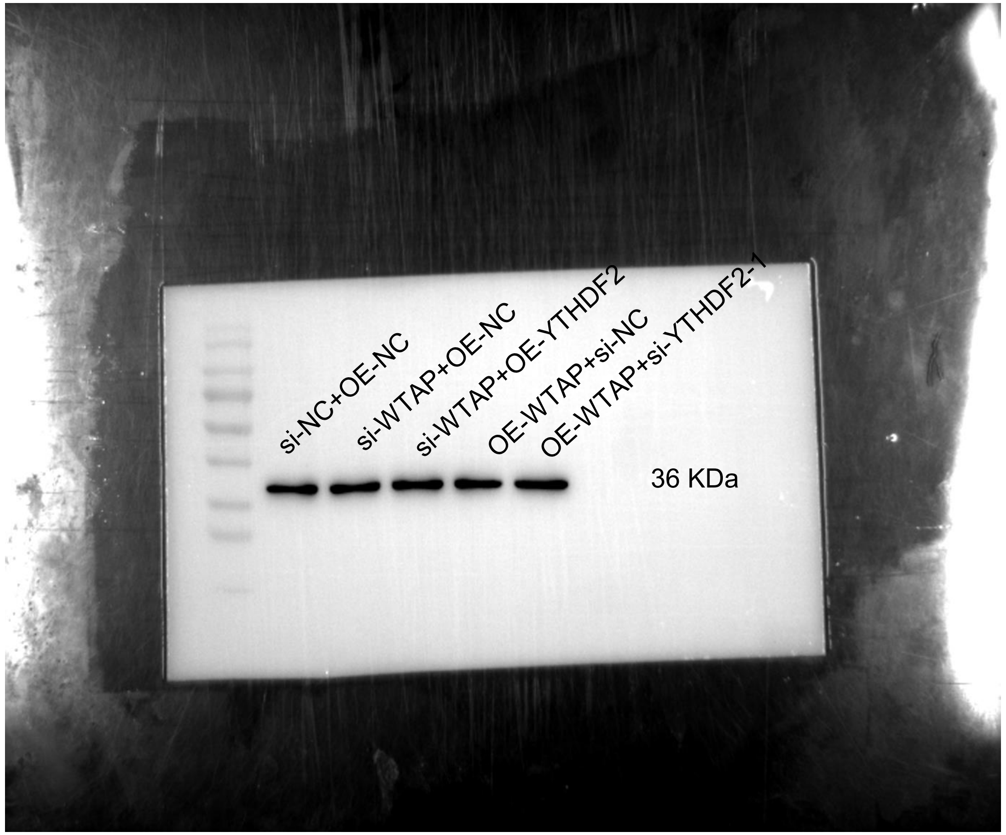

Figure6H GPX4(as shown in this study)

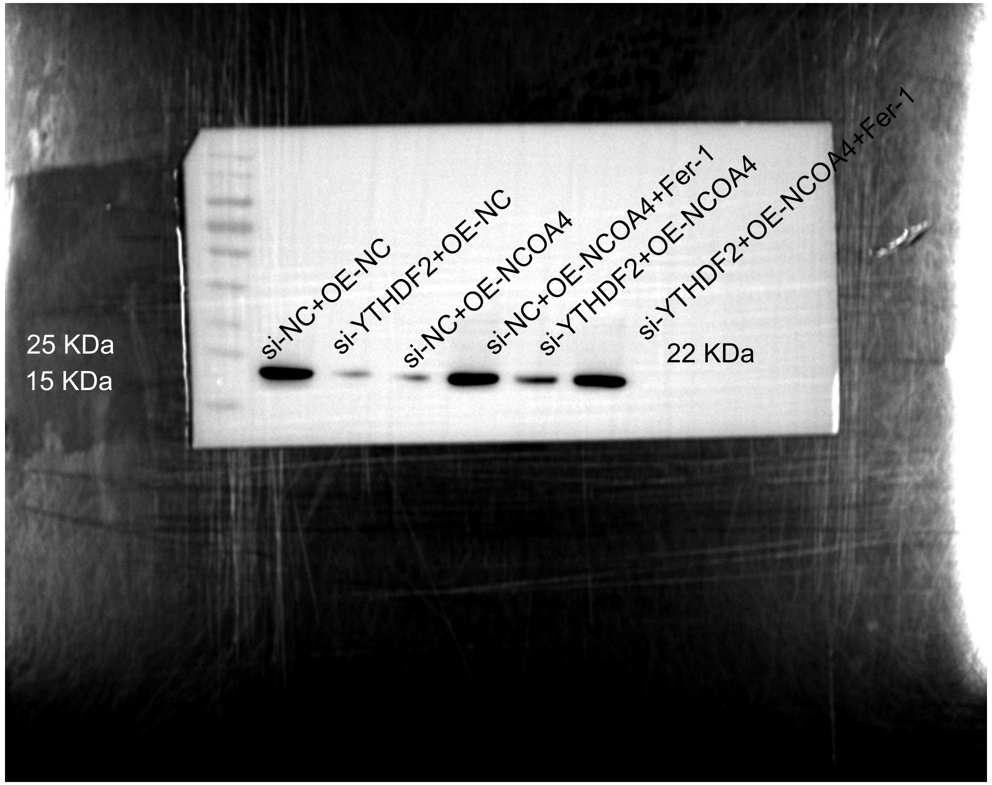

Figure6H NRF2(as shown in this study)

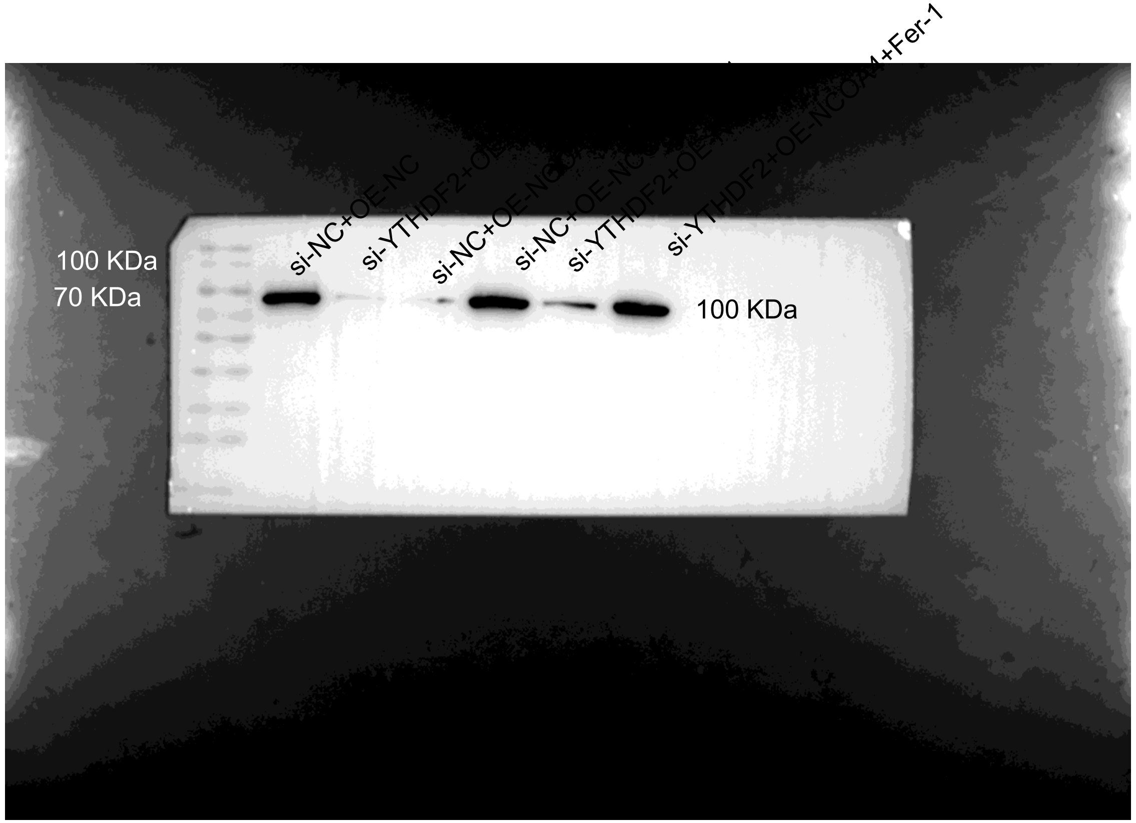

Figure6H GAPDH(as shown in this study)

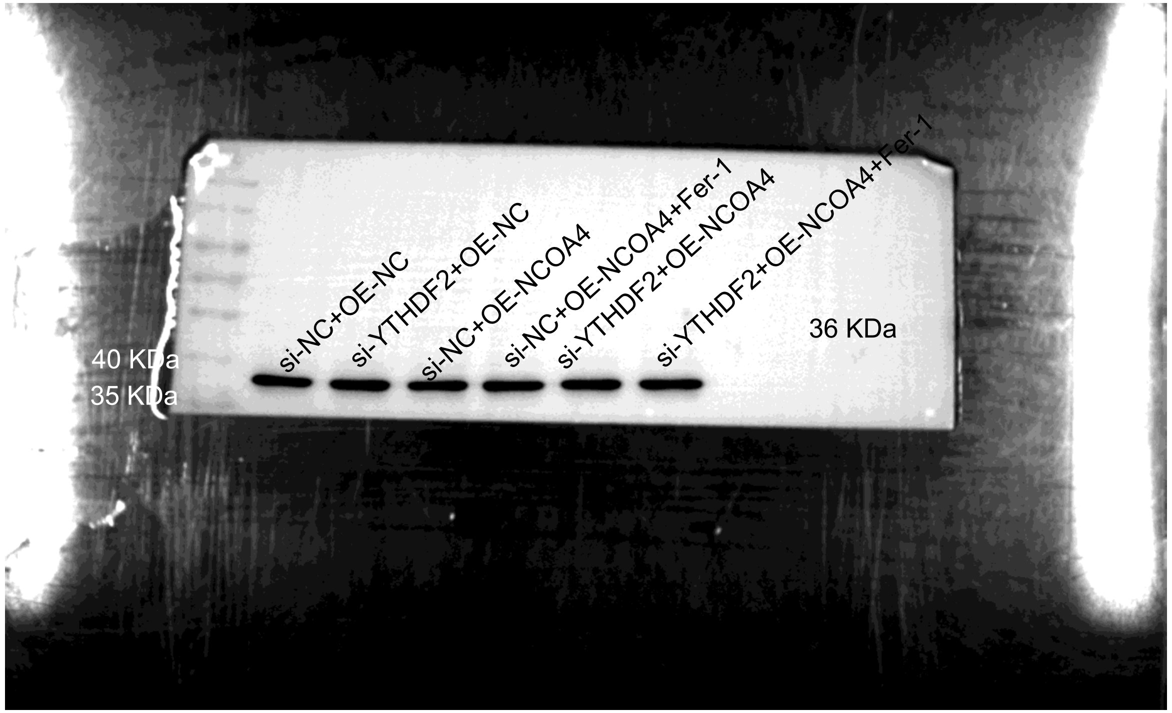

Figure7K Gpx4-1(as shown in this study)

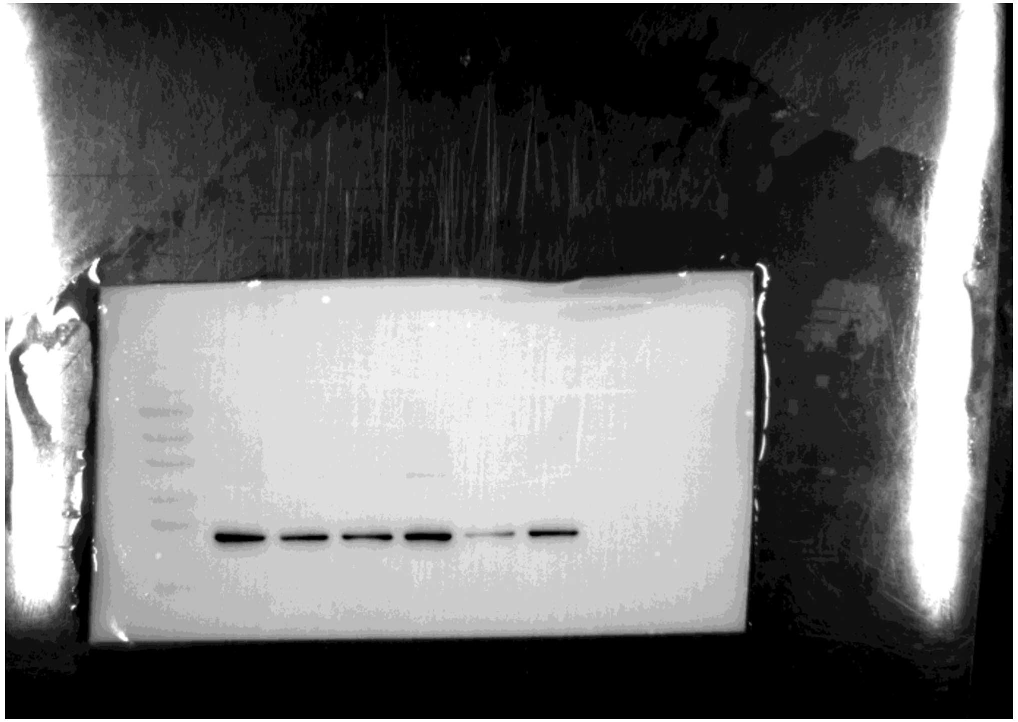

Figure7K Gpx4-2

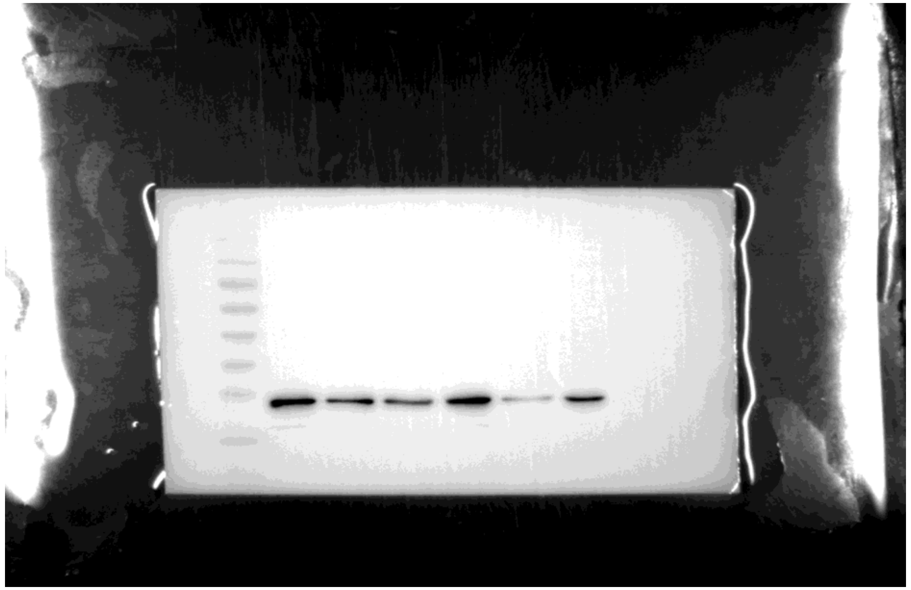

Figure7K Gpx4-3

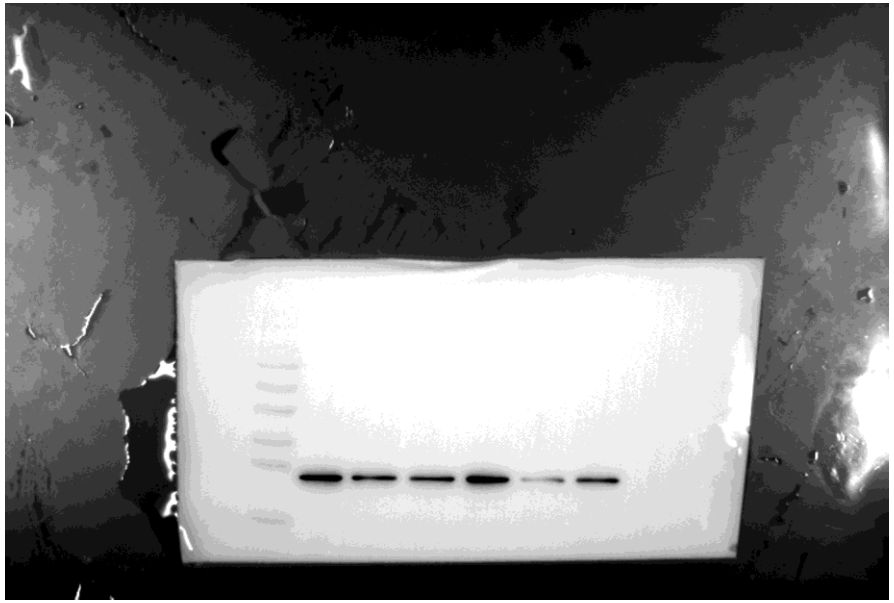

Figure7K Nrf2-1(as shown in this study)

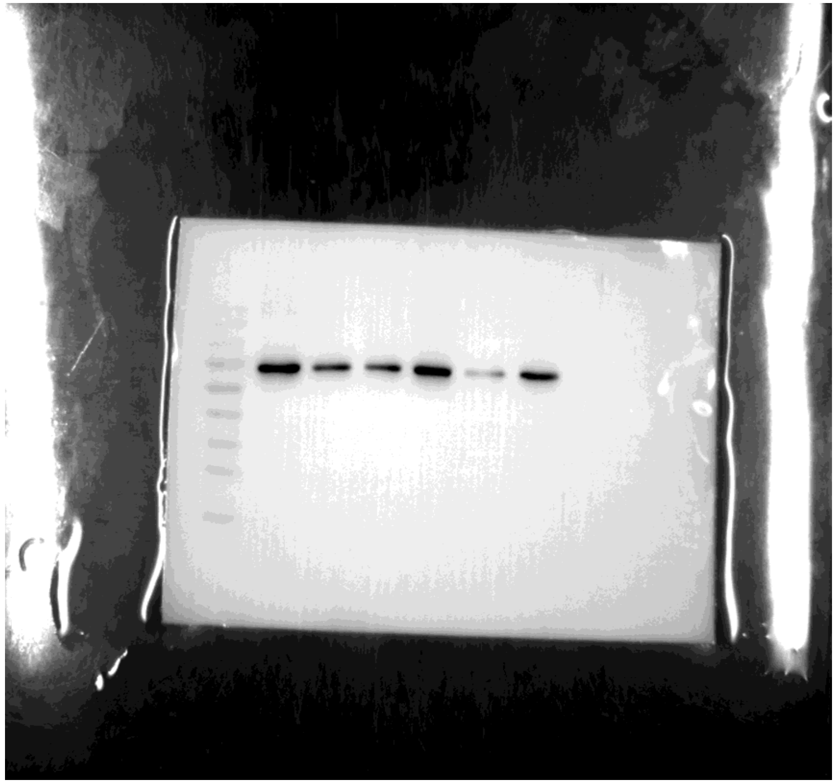

Figure7K Nrf2-2

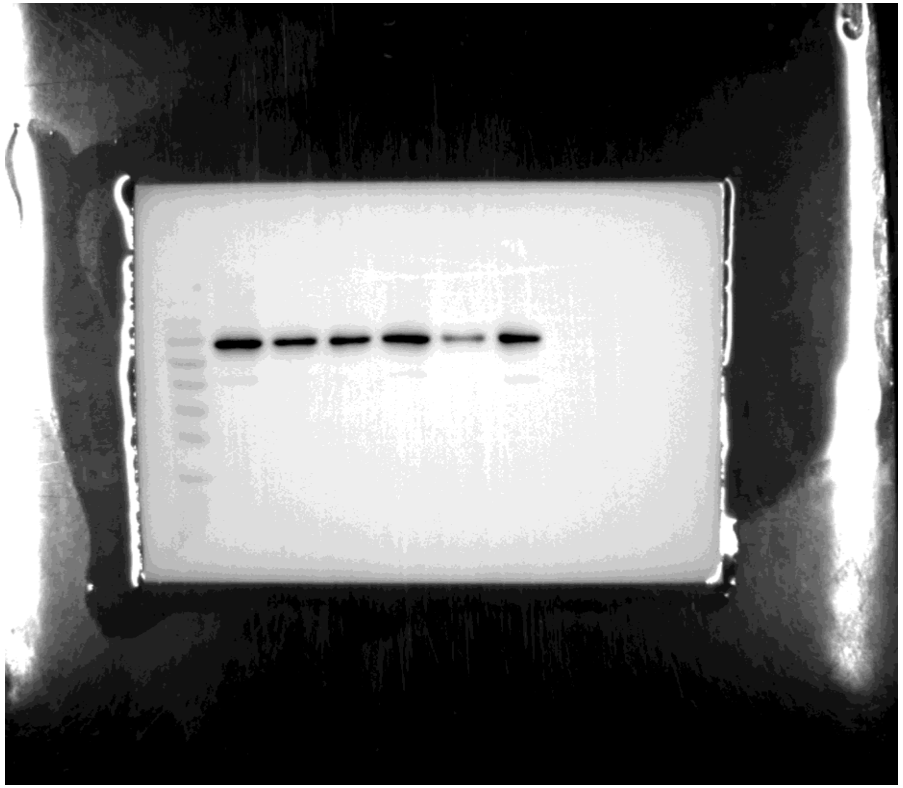

Figure7K Nrf2-3(as shown in this study)

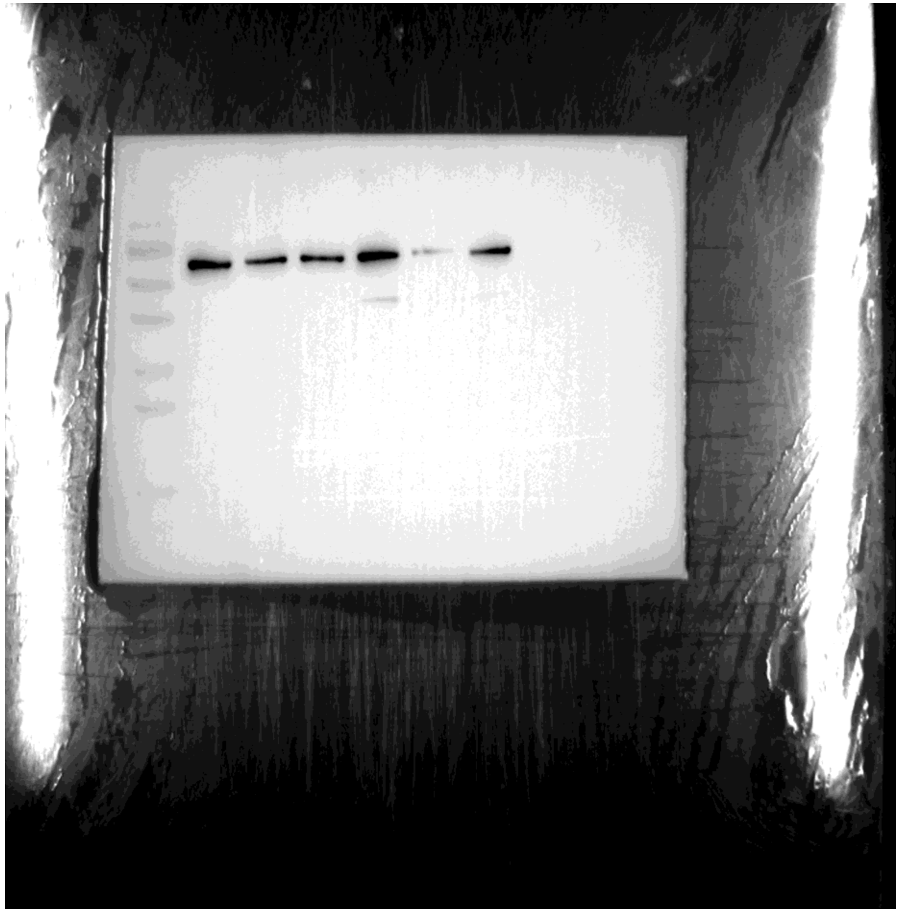

Figure7K GAPDH-1(shown in this study)

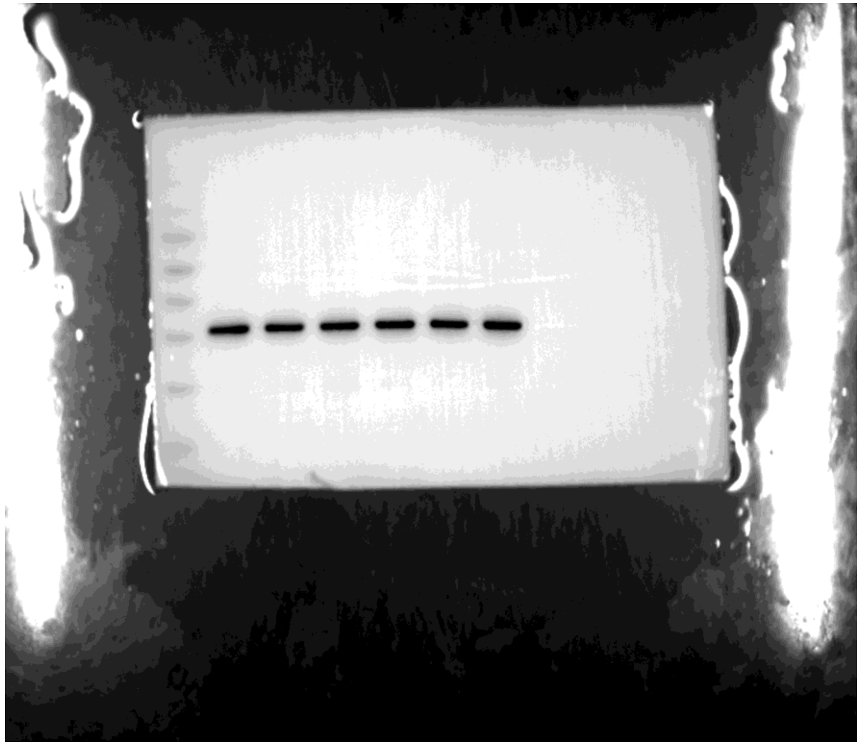

Figure7K GAPDH-2

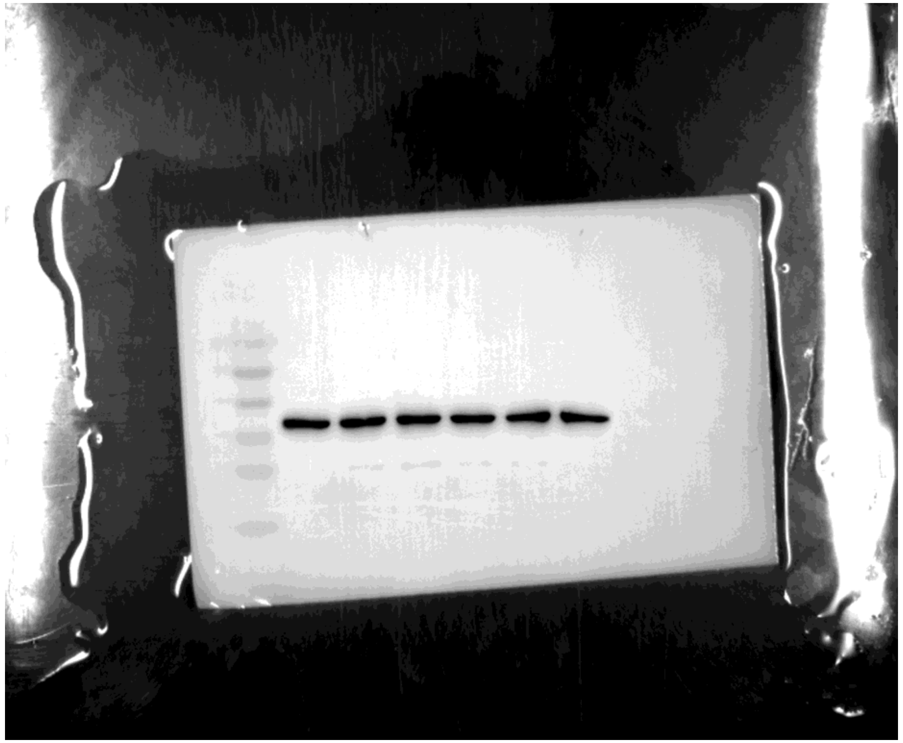

Figure7K GAPDH-3

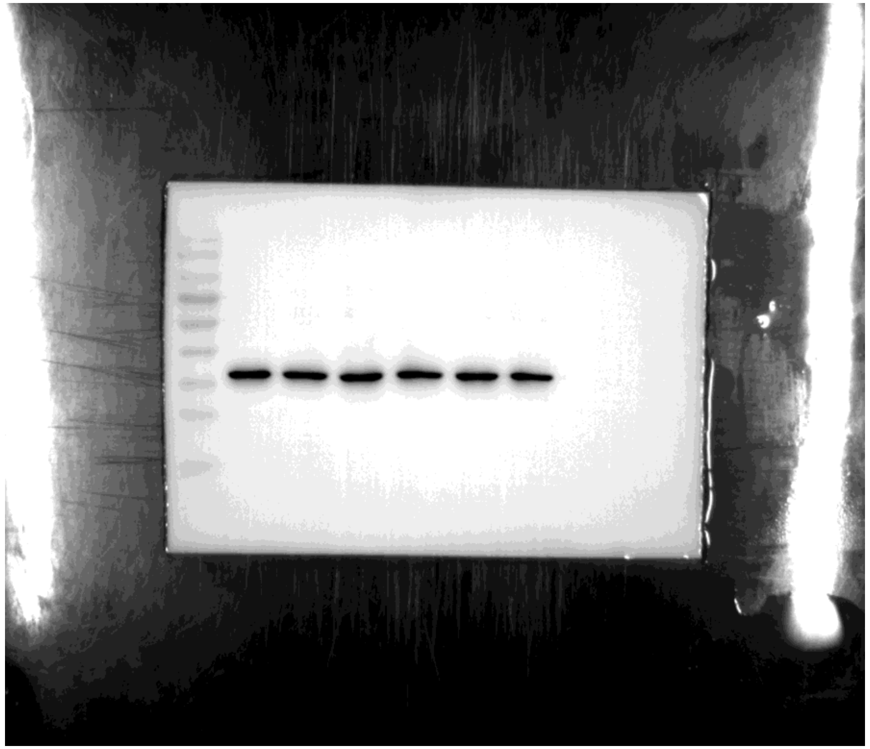

Supplement: Supplementary file 2 — Additional file2 [file 13148_2025_2004_MOESM2_ESM.pdf]
